# Efficacy and safety of camrelizumab plus apatinib during the perioperative period in resectable hepatocellular carcinoma: a single-arm, open label, phase II clinical trial

**DOI:** 10.1136/jitc-2022-004656

**Published:** 2022-04-01

**Authors:** Yongxiang Xia, Weiwei Tang, Xiaofeng Qian, Xiangcheng Li, Feng Cheng, Ke Wang, Feng Zhang, Chuanyong Zhang, Donghua Li, Jinhua Song, Hui Zhang, Jie Zhao, Aihua Yao, Xiaofeng Wu, Chen Wu, Guwei Ji, Xisheng Liu, Feipeng Zhu, Lang Qin, Xuan Xiao, Zhenhua Deng, Xiangyi Kong, Si Li, Yangyang Yu, Wenjing Xi, Wanglong Deng, Chuang Qi, Hanyuan Liu, Liyong Pu, Ping Wang, Xuehao Wang

**Affiliations:** 1 Hepatobiliary Center, The First Affiliated Hospital of Nanjing Medical University, Key Laboratory of Liver Transplantation, Chinese Academy of Medical Sciences, NHC Key Laboratory of Living Donor Liver Transplantation, Nanjing, China; 2 Department of Radiology, The First Affiliated Hospital of Nanjing Medical University, Nanjing, China; 3 Department of Pathology, The First Affiliated Hospital of Nanjing Medical University, Nanjing, China; 4 The State Key Laboratory of Translational Medicine and Innovative Drug Development, Jiangsu Simcere Diagnostics Co, Nanjing, China

**Keywords:** antibodies, neoplasm, antineoplastic protocols, immunity, immunotherapy, liver neoplasms

## Abstract

**Objective:**

This study aimed to assess the efficacy and safety of camrelizumab plus apatinib in patients with resectable hepatocellular carcinoma (HCC) as neoadjuvant therapy.

**Methods:**

Initially, 20 patients with HCC were screened and 18 patients with resectable HCC were enrolled in this open-label, single-arm, phase II clinical trial. Patients received three cycles of neoadjuvant therapy including three doses of camrelizumab concurrent with apatinib for 21 days followed by surgery. Four to 8 weeks after surgery, patients received eight cycles of adjuvant therapy with camrelizumab in combination with apatinib. Major pathological reactions (MPR), complete pathological reactions (pCR), objective response rate (ORR), relapse-free survival (RFS), and adverse events (AE) were assessed. In addition, cancer tissue and plasma samples were collected before and after treatment, and genetic differences between responding and non-responding lesions were compared by tumor immune microenvironment (TIME) analysis, circulating tumor DNA (ctDNA) analysis and proteomics analysis.

**Results:**

In 18 patients with HCC who completed neoadjuvant therapy, 3 (16.7%) and 6 (33.3%) patients with HCC reached ORR based on Response Evaluation Criteria in Solid Tumors (RECIST) V.1.1 and modified RECIST criteria, respectively. Of the 17 patients with HCC who received surgical resection, 3 (17.6%) patients with HCC reported MPR and 1 (5.9%) patient with HCC achieved pCR. The 1-year RFS rate of the enrolled patients was 53.85% (95% CI: 24.77% to 75.99%). Grade 3/4 AEs were reported in 3 (16.7%) of the 18 patients, with the most common AEs being rash (11.1%), hypertension (5.6%), drug-induced liver damage (5.6%), and neutropenia (5.6%) in the preoperative phase. The 289 NanoString panel RNA sequencing showed that TIME cell infiltration especially dendritic cells (DCs) infiltration was better in responding tumors than in non-responding tumors. Our results of ctDNA revealed a higher positive rate (100%) among patients with HCC with stage IIb–IIIa disease. When comparing patients with pCR/MPR and non-MPR, we observed more mutations in patients who achieved pCR/MPR at baseline (6 mutations vs 2.5 mutations, p=0.025). Patients who were ctDNA positive after adjuvant therapy presented a trend of shorter RFS than those who were ctDNA negative. Proteomic analysis suggested that abnormal glucose metabolism in patients with multifocal HCC might be related to different sensitivity of treatment in different lesions.

**Conclusion:**

Perioperative camrelizumab plus apatinib displays a promising efficacy and manageable toxicity in patients with resectable HCC. DCs infiltration might be a predictive marker of response to camrelizumab and apatinib as well as patients’ recurrence. ctDNA as a compose biomarker can predict pathological response and relapse. Abnormal glucose metabolism in patients with multifocal HCC may be related to different sensitivity of treatment in different lesions.

**Trial registration number:**

NCT04297202.

**Key messages**

- The combination therapy of camrelizumab plus apatinib has been shown to be effective as a first-line and second-line treatment for patients with hepatocellular carcinoma (HCC). In terms of efficacy and safety of its combination as neoadjuvant therapy for patients with HCC, few studies report this issue.
- Perioperative camrelizumab plus apatinib displays a promising efficacy and manageable toxicity in patients with resectable HCC. Dendritic cells infiltration might be a predictive marker of response to camrelizumab and apatinib as well as patients’ recurrence. Circulating tumor DNA as a compose biomarker can predict pathological response and relapse.
- A small dose of neoadjuvant therapy combined with postoperative assistance plays a role in inhibiting HCC recurrence and postoperative adjuvant therapy is very necessary in improving the lowpathological reactions rate brought by low-intensity neoadjuvant therapy.

## Introduction

Liver malignancy, as one of the most common solid tumors, is the second leading cause of cancer-related deaths in the world with estimated new deaths of 830,180 in 2020.^1^ Hepatocellular carcinoma (HCC), as the most common type of primary liver malignancy across the world, shows an increasing prevalence rate globally.^2^ Approximately 40% patients with HCC were in locally advanced stage (CNLC, Chinese Liver Cancer Stage, IIb/IIIa stage) when diagnosed in China, who were not recommended for surgical resection according to the guidelines from European Association for the Study of the Liver and American Association for the Study of Liver Diseases.^3–7^ However, in China, patients with locally advanced HCC who meet the certain criteria (such as more than three tumor nodules in the same segment or lobe and can be controlled by radiofrequency ablation during surgery, completely resected tumor localized in one lobe, obstructive jaundice caused by resectable intrahepatic tumors, portal lymph node metastases which can be managed with additional intraoperative lymph node dissection or postoperative external radiation therapy) are still eligible for surgical resection and may achieve negative surgical margin status.^8^ Unfortunately, even after surgical resection and active postoperative management, 54% of patients developed recurrent HCC at a median of 22 months after primary resection.^9 10^ The emergence of neoadjuvant chemotherapy has been proved to bring more surgical opportunity for patients with cancer with unresectable lesions.^11 12^ While few studies report the perioperative systematic therapy in patients with HCC, developing novel and reliable therapeutic intervention to reduce the recurrence and improve the prognosis of patients with locally advanced HCC is urgent.

Recently, the combination of immunotherapy and anti-angiogenesis therapy is of great interest in the treatment of cancer. Preclinical studies have disclosed a synergistic antitumor effect of immunotherapy plus anti-angiogenesis therapy via regulating multiple signal pathways.^13–16^ For instance, the IMbrave150 study exhibits that atezolizumab plus bevacizumab improves the progression-free survival (PFS) and overall survival in unresectable HCC compared with sorafenib alone.^17^ The KEYNOTE-524 study demonstrated that patients with locally advanced unresectable HCC treated with pembrolizumab plus lenvatinib achieved an objective response rate (ORR) of 46.0% (based on modified Response Evaluation Criteria in Solid Tumors (mRECIST) criteria).^18^ Aforementioned studies have proved the clinical value of immunotherapy plus anti-angiogenesis therapy in patients with unresectable HCC, while as to its role serving as the neoadjuvant therapy in patients with locally advanced HCC, only a few studies report this issue. For instance, nivolumab plus cabozantinib as neoadjuvant therapy in patients with locally advanced HCC presents a margin-negative resection rate of 80% and major pathological response (MPR) rate of 42%.^19^

Camrelizumab is a program-death receptor 1 (PD-1) inhibitor and has been approved by Chinese Food and Drug Administration in HCC.^20^ Meanwhile, apatinib, a highly selective tyrosinase inhibitor acting on vascular endothelial growth factor 2, which is first developed in China, has shown a certain efficacy in patients with advanced HCC.^21 22^ Recently, the combination therapy of camrelizumab plus apatinib as first-line and second-line therapy in patients with HCC has shown a certain efficacy profile. The RESCUE study illuminates an ORR of 34.3% and a PFS of 5.7 months in patients with advanced HCC receiving camrelizumab plus apatinib.^23^ While, as to the efficacy and safety profile of their combination serving as the neoadjuvant therapy in patients with HCC, few studies report this issue.

Our study aimed to evaluate the efficacy and safety of camrelizumab plus apatinib as the neoadjuvant therapy in patients with HCC. In addition, genetic differences between responding and non-responding lesions were compared by tumor immune microenvironment (TIME) analysis, circulating tumor DNA (ctDNA) analysis and proteomics analysis.

## Methods

### Study design and patients

In this single-armed, open-labeled, phase II, prospective study, patients with HCC who were willing to receive camrelizumab plus apatinib as neoadjuvant therapy were recruited between December 2019 and June 2021 in Jiangsu Province Hospital. The main inclusion criteria were as follows: (i) had clinical diagnosis of HCC or had histologically or cytologically confirmed HCC; (ii) age no less than 18 years; (iii) not received systematic therapy for HCC prior to this study; (iv) with at least one measurable lesion as the target lesion according to the Response Evaluation Criteria in Solid Tumors (RECIST) criteria (V.1.1)^24^ (v) China liver cancer staging of IIb or IIIa with potentially resectable lesion; (vi) Eastern Cooperative Oncology Group performance status (ECOG PS) of 0–1. Meanwhile, patients met the following criteria were excluded from the present study: (i) known intrahepatic cholangiocarcinoma (ICC) or mixed HCC, sarcomatoid HCC and hepatic fibrolamellar carcinoma; (ii) malignant tumor except HCC in the past 5 years; (iii) undergoing or have previously received the organ transplantation or allogeneic bone marrow transplantation; (iv) moderate-to-severe ascites with clinical symptoms needed drainage; (v) history of gastrointestinal bleeding, with gastrointestinal bleeding tendency, abdominal fistula, gastrointestinal perforation, or abdominal abscess within 6 months prior to the initiation of this study.

### Treatment

After enrolment, all patients received the neoadjuvant therapy with camrelizumab plus apatinib for three cycles with each cycle lasting for 2 weeks. In details, the camrelizumab was administered intravenously at a dose of 200 mg each time every 2 weeks for three cycles, and the apatinib was taken orally and continuously with a dose of 250 mg for 21 days from the beginning of the study. Then, at the 46 days after initiating administration of camrelizumab, the hepatectomy was performed for those patients with resectable lesions. Subsequently, adjuvant therapy including combination of camrelizumab and apatinib was administrated within 4 weeks after the hepatectomy. In detail, the camrelizumab was administered intravenously at a dose of 200 mg each time, every 3 weeks for eight cycles, the apatinib was taken orally with a dose of 250 mg. During the study, dose discontinuations, interruptions, and modifications of apatinib were permitted until meeting the prespecified criteria for treatment resumption per protocol, in details, the dose discontinuations, interruptions, and modifications in dose frequency of apatinib (to 250 mg/day for 1 day on-1 day off, 5 days on-2 days off, or 7 days on-7 days off) were allowed. Camrelizumab treatment was continued until investigator-assessed disease progression, unacceptable toxicity, withdrawal of consent, investigator decision, or study completion.

### Endpoints and assessments

The primary endpoint was MPR defined as 90%–99% tumor necrosis in resected tissue. The secondary endpoint included the postoperative pathological complete response (pCR) defined as no residual cancer cells in the resected tissue, preoperative ORR which was calculated as complete response (CR) rate plus partial response (PR) rate under CT (based on RECIST V.1.1 and mRECIST criteria),^25^ postoperative relapse-free survival (RFS), adverse events (AEs) during the whole study based on the National Cancer Institute Common Toxicity Criteria for Adverse Events V.5.0^26^ and perioperative complications. Both imaging and pathology were identified by two specialists in their respective fields. The exploratory endpoint included the gene expression profiling of a custom panel including the TIME analysis, the ctDNA analysis and the proteomics analysis.

### 289 NanoString panel RNA sequencing

Tumor tissues were collected pre-neoadjuvant therapy (1–7 days before treatment) and at surgery for gene expression analysis. Gene expression was measured using the nCounter platform (NanoString Technologies, Seattle, USA) and transcriptome analysis was based on the 289-immunogene panel. This panel allows simultaneous analysis of 289 genes involved in the immune response in cancer. For each sample, quality control (QC) indicators include the imaging QC, binding density QC, positive control linearity QC, positive control limit of detection QC, positive normalization factor, and content normalization factor were then calculated. Samples qualified for QC were included in subsequent analysis. The raw data of each sample and gene were standardized against internal External RNA Controls Consortium to eliminate technical variability in the assay, and then counts were normalized to the geometric mean of endogenous housekeeping genes followed by log2 transformation.

### Estimation of TIME cell infiltration

Marker genes of 14 immune cell types, including B-cells, dendritic cells (DCs), macrophages, T-cells, regulatory T cells (Tregs), CD8 T cells, exhausted CD8, neutrophils, mast cells, cytotoxic cells, natural killer (NK) cells, NK CD56dim cells, CD45, and Th1 cells were retrieved from the method previously reported.^27–29^ We further divided the macrophages into macrophages M1 and macrophages M2 according to the previous reports.^30 31^ All TIME cell infiltration scores were calculated as arithmetic mean of the constituent genes.^27^

### Generation of TIME signatures

We constructed a set of gene sets that stored genes associated with some biological processes, including (1) CTL (cytotoxic T lymphocytes) levels; (2) CYT (cytolytic activity) score; (3) chemokines; (4) T cell markers; (5) total TILs (tumor infiltrating lymphocytes) score; (6) Teff (T-effector) score; (7) interferon (IFN)-γ signature; (8) GEP (gene expression profiling) score. The CYT score of each sample can be evaluated based on the geometric mean of the product of PRF1 and GZMA genes^32^; GEP score was calculated as a weighted linear average of the constituent genes^33 34^; the remaining TIME signatures were calculated as arithmetic mean of the corresponding gene.^27^

### Gene set enrichment and pathway analysis

The Kyoto Encyclopedia of Genes and Genomes (KEGG)/gene ontology (GO) enrichment analysis was performed in the ClusterProfiler R package. The list of gene IDs was used as the input file. The Benjamini-Hochberg method was used to adjust the p values. The cut-off of p values was set to 0.05. The enrichment results were visualized with the ggplot2 R package. Enrichment statistic was set to classic.

### ctDNA sequencing and bioinformatics analysis

Plasma samples were collected pre-neoadjuvant (T0, 1–7 days before treatment, baseline), post-neoadjuvant (T1, 1–7 days after treatment), post surgery (T2, 7–10 days after surgery) and post-adjuvant therapy (T3, 1–7 days after treatment) for ctDNA analysis. ctDNA was extracted using the Apostle MiniMaxTM High Efficiency ctDNA Isolation Kit (Apostle, USA) with optimized manufacturer’s protocols. Sequencing libraries were prepared using the KAPA Hyper Prep kit (KAPA Biosystems, USA) according to the manufacturer’s protocol. Libraries were enriched using a panel targeting 202 genes. The target-enriched library was then sequenced on Illumina NovaSeq6000 NGS platforms (Illumina, USA) according to the manufacturer’s instructions. The average sequencing depth of ctDNA samples was ~30,000 X.A custom pipeline was developed to perform reads alignment, variants calling, fusion detection, CNV (copy number variation) identification and QC. The fastp (V.2.20.0)^35^ was used for adapter trimming. Sequence reads were aligned against the human reference genome (hg19) using BWA-mem (V.0.7.17)^36^ with additional realignment of select regions using the ABRA2(V.2.21).^37^ Candidate tumor specific mutations, consisting of point mutations, small insertions, and deletions were identified and annotated using VarDict (V.1.5.7)^38^ and InterVar.^39^ CNVs and fusions were analyzed by CNVkit (dx1.1)^40^ and factera (V.1.4.4),^41^ respectively. Additional filter and inspection of somatic mutations, CNVs and fusion results were analyzed by custom scripts. Plasma samples with detectable somatic variants (≥1) were defined as ctDNA positive. Status of ctDNA and its variation trend along with intervention were analyzed to explore their association with pathological response and recurrence.

### Proteomics

Cell pellets/tissue were suspended on ice in 200 µL lysis buffer. Cells/tissue were disrupted with agitation using a homogenizer, and boiling for 5 min. The samples were further ultrasonicated and boiling again for another 5 min. Undissolved cellular debris were removed by centrifugation at 16,000 rpm for 15 min. The supernatant was collected and quantified with a BCA Protein Assay Kit (Bio-Rad, USA). Digestion of protein (200 µg for each sample) was performed according to the filter-aided sample preparation(FASP) procedure described by previous study.^42^ Peptides were labeled with TMT (tandem mass tag) reagents according to the manufacturer’s instructions (Thermo Fisher Scientific, USA). Each aliquot was reacted with one tube of TMT reagent, respectively. After the sample was dissolved in 100 µL of 0.05M tetraethylammonium bromide(TEAB)solution, pH 8.5, the TMT reagent was dissolved in 41 µL of anhydrous acetonitrile. The mixture was incubated at room temperature for 1 hour. Then 8 µL of 5% hydroxylamine to the sample and incubate for 15 min to quench the reaction. The multiplex labeled samples were pooled together and lyophilized. TMT-labeled peptides mixture was fractionated using a Waters XBridge BEH130 column on Agilent 1290 high performance liquid chromatography(HPLC) operating at 0.3 mL/min. Buffer A consisted of 10 mM ammonium formate and buffer B consisted of 10 mM ammonium formate with 90% acetonitrile; both buffers were adjusted to pH 10 with ammonium hydroxide. The fractions were dried for nano LC-MS analysis.

### Statistical analysis

SPSS V.26.0 (IBM Corp), GraphPad Prism V.6.0 (GraphPad Software) and R system were applied for data analysis and graph illustrating. The unpaired t-test was used for comparisons between two-group means, where the data could be assumed to have been sampled from populations with normal (or approximately normal) distributions. Survival analyses were performed with Kaplan-Meier curves and log-rank test, and p value<0.05 was used as significant threshold in the remaining statistical analysis.

## Results

### Study flow

**Figure 1:**
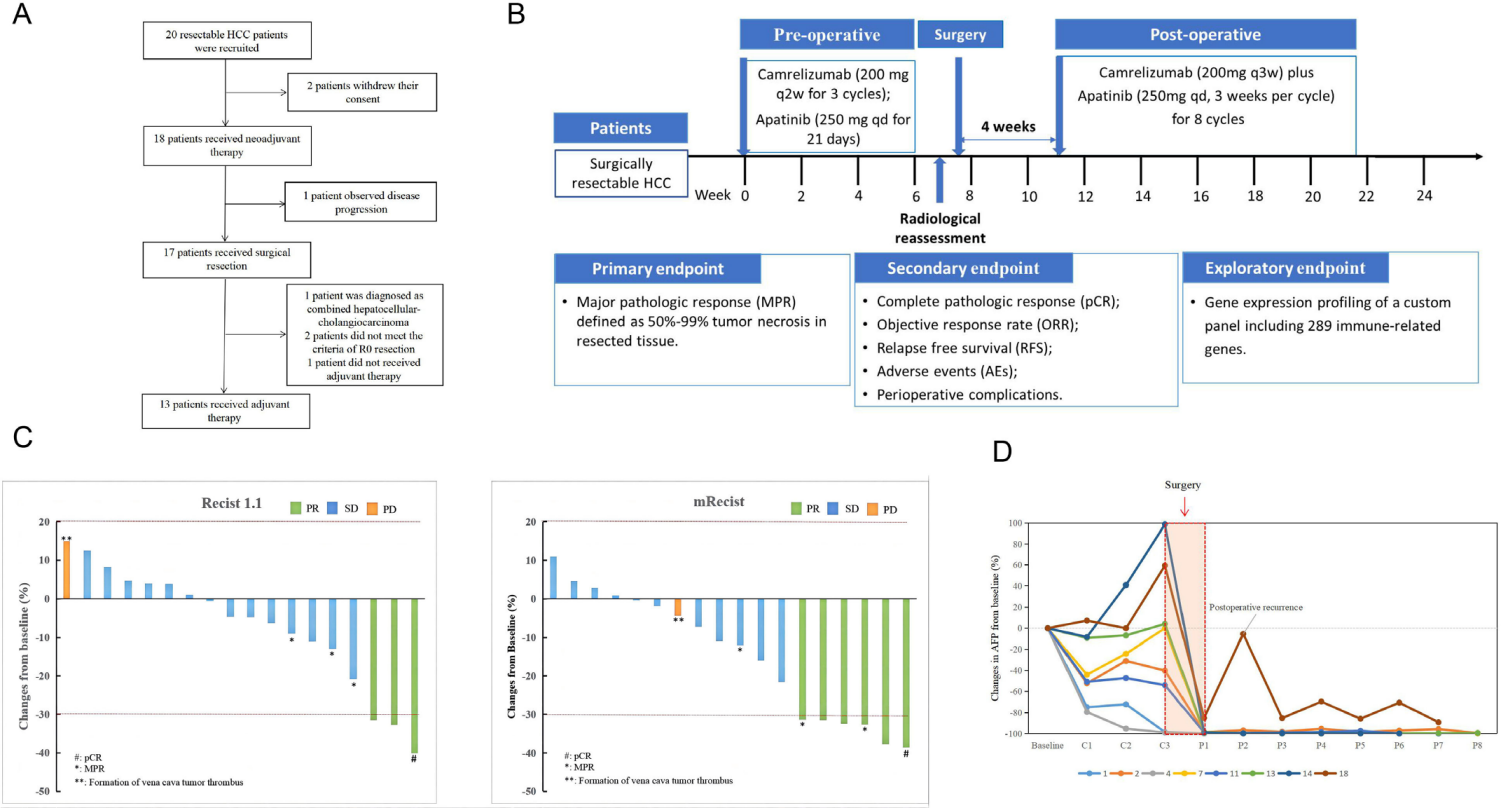
Study design. (A) Flowchart of enrolled patients. (B) Flowchart of therapeutic regimen. (C) Treatment was evaluated for the enrolled patients based on RECIST V.1.1 criteria and mRECIST criteria. (D) Measuring of AFP level change during perioperative period in patients with surgical resectable HCC. HCC, hepatocellular carcinoma; mRECIST, modified RECIST; RECIST, Response Evaluation Criteria in Solid Tumors.

Totally, 20 patients with resectable HCC were initially recruited (figure 1A). While 2 patients withdrew their consent, 1 patient had disease progression and was unable to receive surgery, the remaining 17 patients received surgical resection. Subsequently, 1 patient was diagnosed with combined hepatocellular-cholangiocarcinoma, and 3 patients violated the study protocol, leaving 13 patients with HCC who received adjuvant therapy. After enrolment, all patients received the neoadjuvant therapy including administration of camrelizumab plus apatinib for three cycles with each cycle lasting for 2 weeks. Then, hepatectomy was performed for patients with resectable lesions 46 days after initiating administration of camrelizumab, adjuvant therapy with a combination of camrelizumab and apatinib was administrated within 4 weeks after hepatectomy (figure 1B).

### Patients’ characteristics

The median age of patients with resectable HCC was 54.7 (range: 34–76) years consisting of 17 (94.4%) men (online supplemental table S1). The ECOG PS score was 0 in 18 (100%) patients with HCC. Moreover, 5 (27.8%) and 13 (72.2%) patients with HCC were classified as Barcelona clinic liver cancer (BCLC) stage B and stage C, respectively. Other detailed baseline characteristics were shown in online supplemental table S1.

10.1136/jitc-2022-004656.supp1 Supplementary data

### Treatment response

In 18 patients with HCC who completed neoadjuvant therapy, 3 (16.7%) patients with HCC reached ORR based on RECIST V.1.1 criteria and 6 (33.3%) patients with HCC achieved ORR based on mRECIST criteria (figure 1C, online supplemental table S2). Among the 17 patients with HCC who underwent surgical resection, 3 (17.6%) patients with HCC achieved MPR, 1 patient with HCC (5.9%) achieved pCR and the remaining 13 patients were non-MPR (online supplemental table S3).

The alpha-fetoprotein(AFP) level and its longitudinal change during perioperative period in surgical resectable HCC could provide some evidence of early tumor recurrence and tumor residues. The AFP level was determined in 17 patients with HCC at study baseline, preoperative and postoperative period. Nine (52.9%) patients had AFP levels <400 µg/L, and 8 (47.1%) patients had AFP levels ≥400 µg/L. Among eight patients with abnormal baseline AFP level, two patients experienced AFP level decline ranging from 54% to 99% after neoadjuvant therapy, all patients experienced AFP level decline ranging from 85% to 100% after surgical resection (figure 1D).

**Figure 2:**
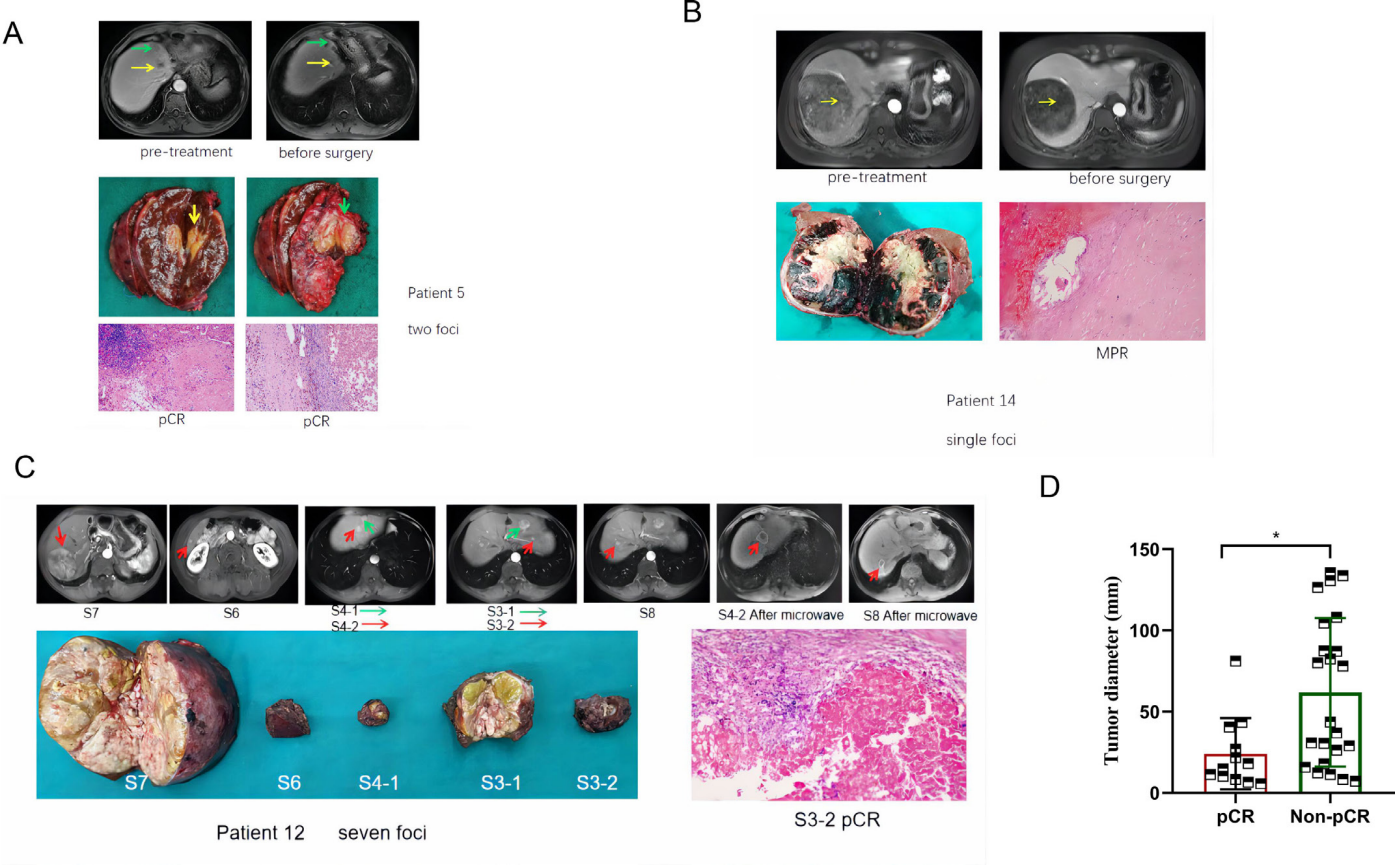
Response to treatment in typical cases. (A) Patient 5; male; 70 years old; stage IIb; total five lesions, maximum diameter 32.6 mm; after neoadjuvant therapy, RECIST V.1.1 and mRECIST evaluation were PR; surgical pathology only showed two large lesions and were both pCR. (B) Patient 14; female; 53 years old; stage IIIa; tumor diameter 108.1 mm; after neoadjuvant therapy, RECIST V.1.1 evaluation was stable disease, mRECIST evaluation was PR; surgical pathology was MPR. (C) Patient 12; male; 55 years old; stage IIb; seven tumor nodules; the largest of which was 80 mm in diameter; after neoadjuvant therapy, five tumors were resected, among which S3–2 lesion was treated with pCR, S4–2 and S8 lesions with microwave. DFS is 293 days. (D) The relationship between tumor diameter and pathological response. *P＜0.05. Arrows represent lesions.RECIST, Response Evaluation Criteria in Solid Tumors; mRECIST, modified RECIST;PR, partial response; pCR, pathological reactions;MPR, major pathological reactions; DFS, Disease Free Survival.

In figure 2A–C, we showed the imaging and pathological data of several typical patients. After neoadjuvant therapy, patient five obtained PR according to both RECIST V.1.1 and mRECIST criteria, but pCR was obtained for both pathological scores. The RECIST V.1.1 score of patient 14 was stable disease (SD), but the mRECIST score was PR, and the single foci was MPR. It is worth mentioning that patient 12 had a total of 7 tumor nodules, the largest of which was 80 mm in diameter. After neoadjuvant therapy, five tumors were removed, among which S3–2 lesion gained pCR, S4–2 and S8 lesions were treated with microwave. Imaging evaluations of other patients were shown in the online supplemental figure S1. In particular, we would like to emphasize that patient 17, who was rated progressive disease after neoadjuvant evaluation. Due to the formation of cancer thrombus in the vena cava, surgery was not performed, and radiotherapy was added on the basis of camrelizumab +apatinib. The thrombus disappeared, and transcatheter arterial chemoembolization was continued, and the tumor also shrank, which was evaluated as PR. Surgery was available, but the patient refused surgery and continued with systematic treatment (online supplemental figure S2).

In addition, we evaluated the relationship between tumor diameter and pathological response. To our delight, tumor diameter was significantly higher in the non-pCR group than in the pCR group (figure 2D), suggesting that patients with smaller tumor diameter benefit more from neoadjuvant therapy.

### Safety

Eighteen patients who received neoadjuvant therapy were included in the safety analysis (online supplemental table S4). During the neoadjuvant period, 16 (88.9%) patients had at least one treatment-related adverse event (TRAE). The most common TRAEs of any grade were fever (38.9%), hypertension (33.3%), elevated lactate dehydrogenase (33.3%), and abdominal pain (33.3%) *et al*. Grade 3 or greater TRAEs were reported in 3 (16.7%) of the 18 patients, with the most common AEss being rash (11.1%), hypertension (5.6%), drug-induced liver damage (5.6%), and neutropenia (5.6%). Apatinib administration was suspended in one patient (5.6%) due to elevated blood pressure and resumed after hypertension recovered to grade I. There were two patients requiring glucocorticoids for the management of serious preoperative toxicity events, in details, one patient required glucocorticoid due to severe liver function injury and the other one required glucocorticoid due to severe skin rash. After being treated with corticosteroids in these two patients, their disease remained stable and no disease progress occurred.

The characteristics of surgical and postoperative features were as shown in online supplemental table S5. Seven patients had post-hepatectomy liver failure and classified grade A according to the International Study Group of Liver Surgery, one patient had precordial pain with chest tightness and high-sensitivity troponin increased significantly 6 days after surgery, three patients experienced postoperative biliary leakage, two patients had anemia and treated with blood transfusion therapy. No death occurred during the perioperative period. Furthermore, no complications requiring reoperation occurred.

During the adjuvant treatment period, all patients who received camrelizumab in combination with apatinib had at least one TRAE (online supplemental table S6). The most common TRAEs of any grade were hypertension (61.5%), leukopenia (53.8%), proteinuria (46.2%), and elevated thyroid stimulating hormone (46.2%), *et al*. Grade 3 or greater TRAEs were reported in 5 (38.5%) of the 13 patients, with the most common AEs being hypertension (23.1%), elevated alkaline phosphatase (7.7%), increased glutamyl transferase (7.7%), and diarrhea (7.7%). Seven patients (53.8%) experienced dose adjustment with a manner of 1 day-on 1 day-off. Apatinib administration was suspended in six patients (40%) due to periodontal disease, oral mucositis, blood bilirubin increased, and other reasons, while the apatinib was resumed after those patients recovered from the adverse reactions.

### Patient follow-up

We followed-up 13 patients who underwent R0 resection and completed all the treatments, of them 8 had single lesions and 5 had multiple lesions. There was no recurrence in six (75%) of eight single foci patients and one (20%) of five multi-foci patients. As shown in online supplemental figure S3A, the 1 year RFS rate was 53.85% (95% CI: 24.77% to 75.99%). Online supplemental figure S3A showed that the RFS of patients with MPR/pCR was higher than that of patients without non-MPR, but there was no statistical difference. The RFS of patients with multiple foci was shorter than that of patients with single foci (online supplemental figure S3C). Although the p value was very close to 0.05, it still failed to meet the standard, possibly due to our small sample size.

Specifically, three (37.5%) of the eight patients with a single lesion achieved MPR, one of the three patients relapsed within 1 year, and the other two patients had not relapsed so far. The RFS was 555 and 402 days, respectively. One of the five patients without MPR relapsed, and RFS of the remaining four patients was as follows: 449, 430, 411 and 380 days. For five cases of multiple lesions, only one case of patients with multiple lesions reached pCR, the remaining four patients were non-pCR. Only the patients with all lesions having reached pCR had not relapsed so far, and the RFS was 421 days, while the other four patients with multiple lesions had relapsed within 1 year. Therefore, multiple lesions are more likely to relapse than single lesions. It is possible that postoperative recurrence can be reduced only when all lesions in multiple lesions reach pCR or MPR. In addition, only two (25%) of the eight patients with single lesions have recurrence, which was obviously lower than the recurrence rate of patients who did not receive treatment.

### Pre-neoadjuvant TIME between responding and non-responding lesions

**Figure 3:**
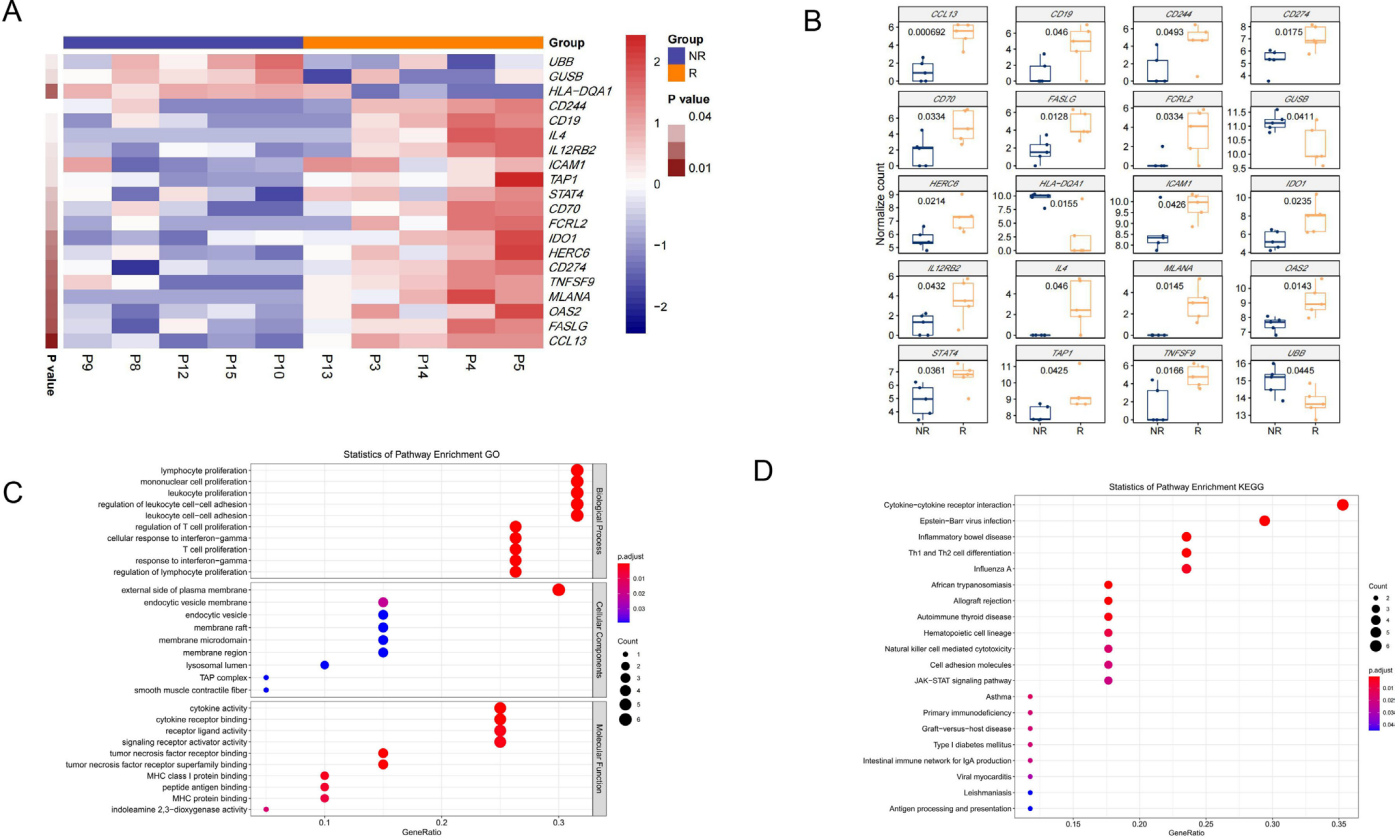
Comparison of expression of immune-related genes between responding and non-responding lesions. (A–B) Expression of immune-related genes per pretreatment sample of response and non-response groups. In the heatmap (A), red indicates an increased gene expression and blue indicates a decreased gene expression. (C–D) GO (C) and Kyoto Encyclopedia of Genes and Genomes (D) pathway analysis of the pretreatment samples. The count represents the number of genes in each pathway and dot size corresponds to ‘count’. GO, gene ontology.

**Figure 4:**
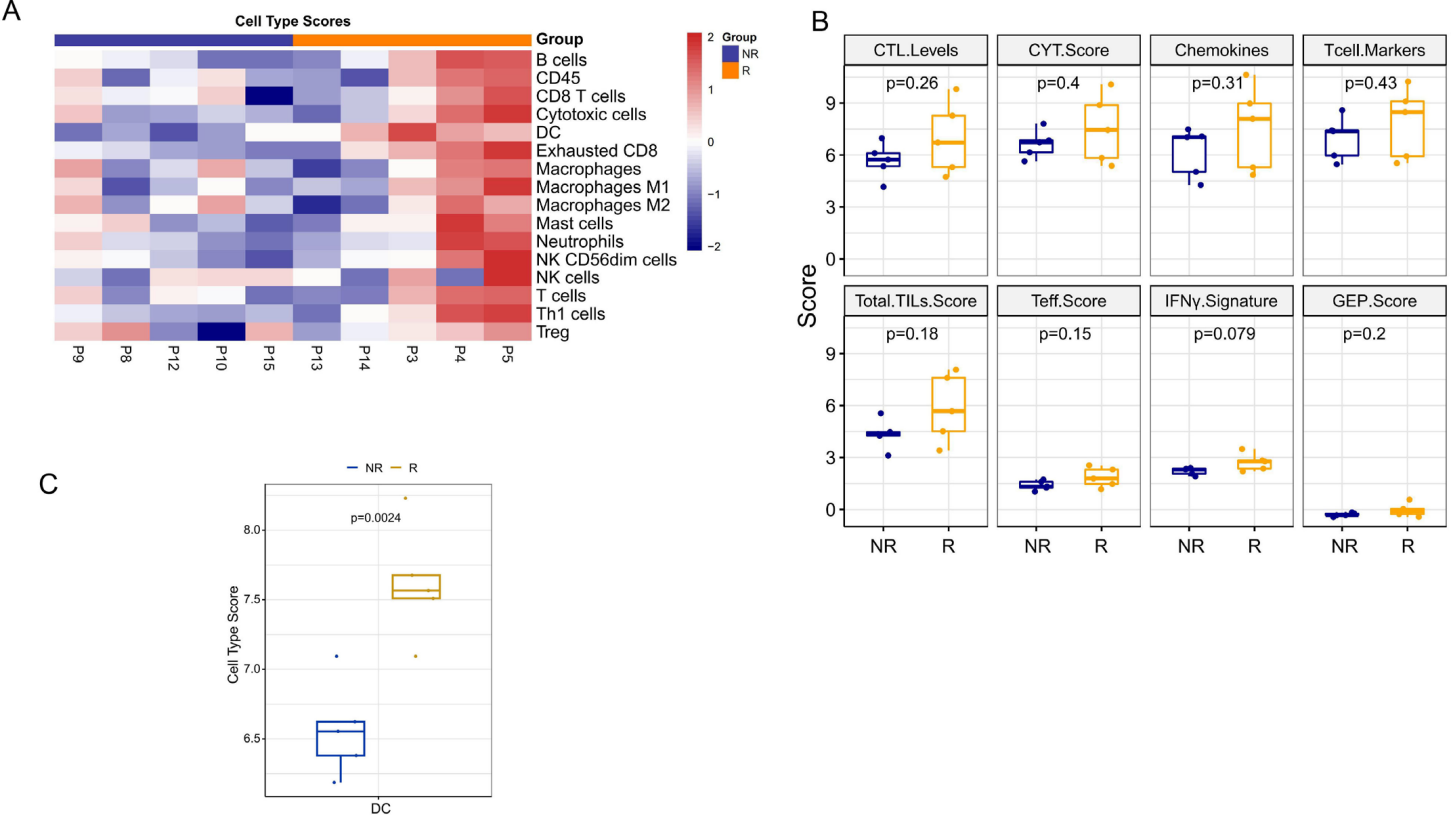
Comparison of cell type scores between responding and non-responding lesions. (A–B) Different cell type scores in per pretreatment sample of response and non-response groups. Red indicates an increased cell type scores and blue indicates a decreased cell type scores. (C) The cell type scores of DCs between response and non-response groups. CTL, cytotoxic T lymphocyte; CYT, cytolytic activity; DC, dendritic cell; GEP, gene expression profiling; IFN, interferon; NK, natural killer; Teff, T-effector; TIL, tumor infiltrating lymphocytes.

The transcriptional profiling of baseline tissue samples was performed to compare responding (≥50% tumor necrosis) (R) and non-responding tumors (<50% tumor necrosis) (NR) to find possible pretreatment biomarkers which can be predictive of the neoadjuvant therapy response. Comparing with non-responding tumors, biopsies in responding tumors revealed a higher transcriptional level of *CCL13, MLANA, TNFSF9, IDO1, CD70, IL12RB2, CD19* and *IL4* as well as a lower transcriptional levels of *HILA-DQ1* and *GUSB* (figure 3A, B). GO terms analysis and KEGG pathways analysis were conducted to identify the biological function of the changed genes between the two groups. The results of the GO analysis revealed that genes were significantly enriched in ‘lymphocyte proliferation’, ‘mononuclear cell proliferation’ and so on (figure 3C). KEGG pathway analysis revealed that genes were highly associated with ‘cytokine-cytokine receptor interaction’, ‘Epstein-Barr virus infection’ and so on (figure 3D). Meanwhile, different TIME cell infiltration score of baseline tumor specimen was analyzed. Totally, there was a better TIME cell infiltration in responding tumors compared with non-responding tumors (figure 4A, B). Especially, the score of DCs was significantly higher in responding tumors (figure 4C). However, TIME signature scores were similar in both groups. The IFN-γ score was higher in responding tumors with no statistical difference (figure 4B). These results indicated that DCs could act as the predictive biomarkers of neoadjuvant therapy in HCC, which needs to be confirmed in further study.

### Changes of TIME in HCC after neoadjuvant therapy of camrelizumab and apatinib

**Figure 5:**
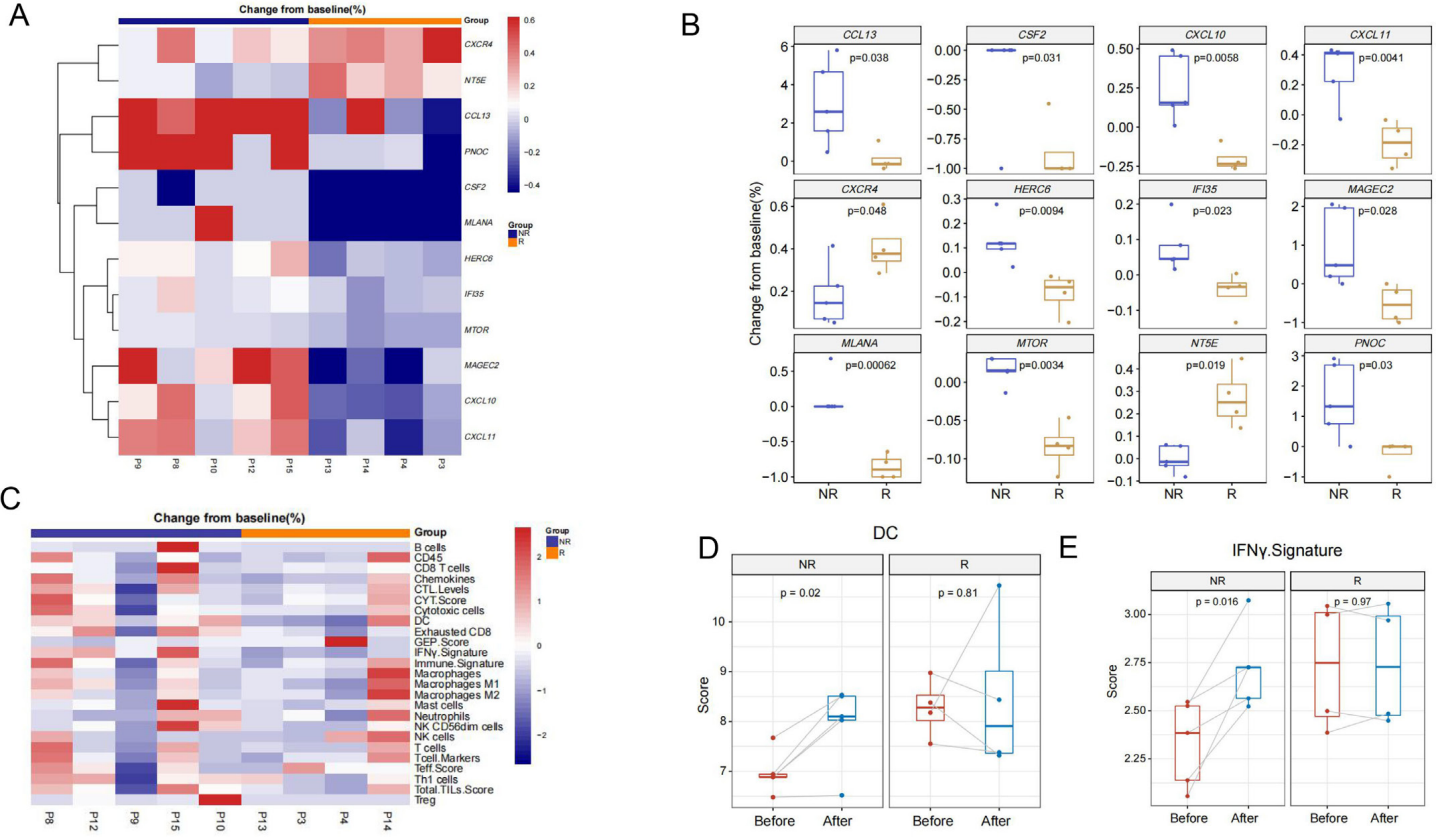
Pre-to-post-treatment changes of tumor immune microenvironment between responding and non-responding lesions. (A–B) Pre-to-post-treatment changes of immune-related genes expression according to response and non-response groups. In the heatmap (A), red indicates an increase and blue indicates a decrease. (C) Pre-to-post-treatment changes of cell type scores and immune signature scores according to response and non-response groups. Red indicates an increase and blue indicates a decrease. (D) Pre-to-post-treatment changes of IFN-γ signature scores between response and non-response groups. (E) Pre-to-post-treatment changes of DCs scores between response and non-response groups. CTL, cytotoxic T lymphocyte; CYT, cytolytic activity; DC, dendritic cell; GEP, gene expression profiling; IFN, interferon; NK, natural killer; Teff, T-effector; TIL, tumor infiltrating lymphocytes.

The gene expression changes of pre/post treatment were also analyzed in our study (figure 5A). Post-treatment assessment revealed increases of *CCL13, CSF2, CXCL10* and so on in non-responding tumors, while increases of *CXCR4, NT5E* and so on in responding tumors (figure 5B). In addition, TIME cell infiltration and TIME signatures were evaluated (figure 5C). The significant post-treatment increases in DCs infiltration and IFN-γ score were observed in non-responding tumors, but non-significant changes in responding tumors (figure 5D, E).

We further explored the relationship between the TIME signature of the post-treatment samples and the recurrence risk. For patients with a single lesion, the risk of recurrence was not associated with IFN-γ in the post-treatment samples (online supplemental figure S4A). Although the DCs of the non-recurrence group was higher than that of the recurrence group, there was no statistical difference in p values, perhaps due to the small sample size (online supplemental figure S4B). However, when we included GEP data of the worst response lesion in patients with multiple focal, the DCs of the non-recurrence group was significantly higher than that of the recurrence group, and there was a statistical difference (online supplemental figure S4C). But, there was still no difference in IFN-γ expression between the two groups (online supplemental figure S4D). These results indicate that the expression of DCs after neoadjuvant therapy might predict the recurrence of patients.

### Association between ctDNA and pathological response

**Figure 6:**
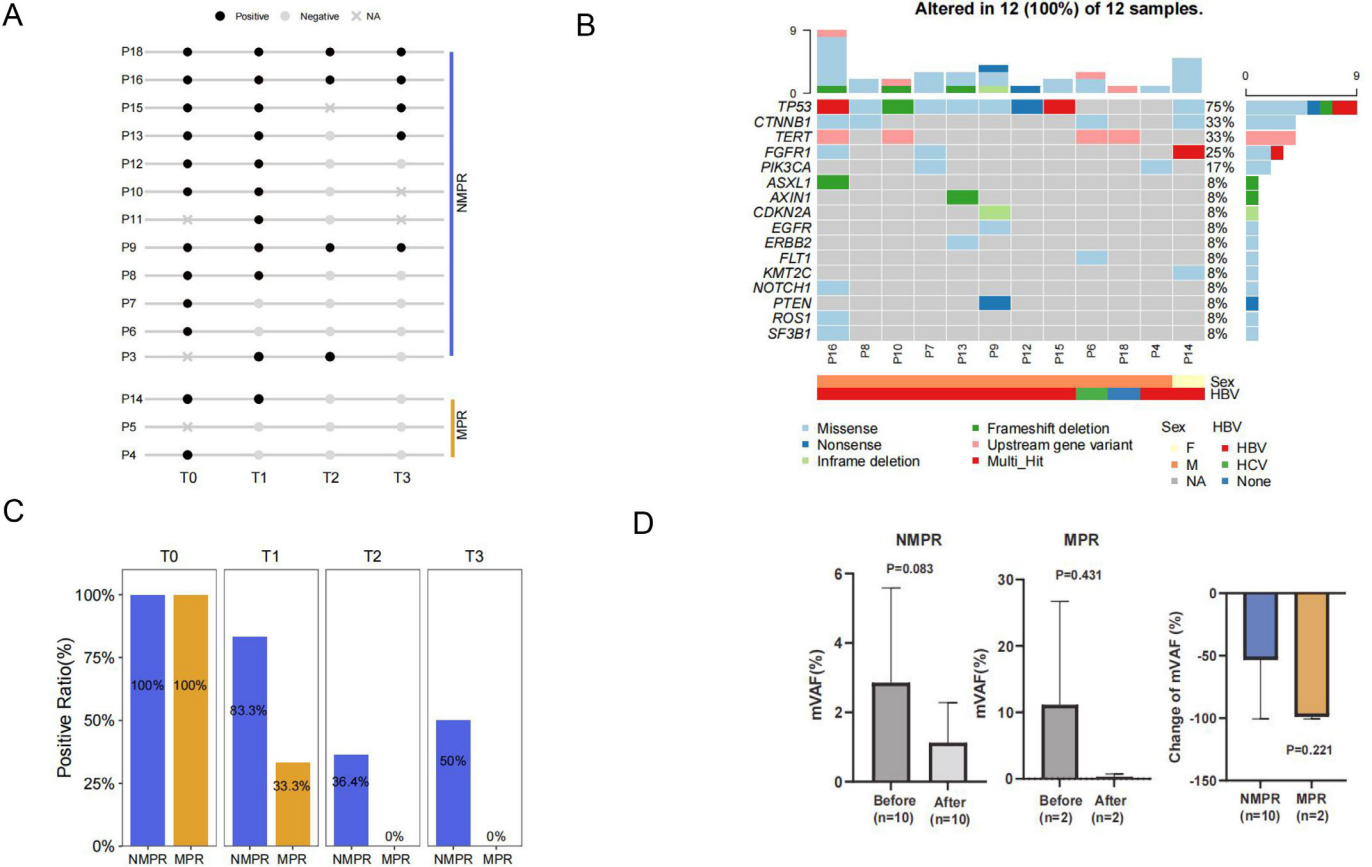
Detection of ctDNA and its association with pathologic response. (A) Plot of ctDNA status for group MPR and non-MPR at T0 (pre-neoadjuvant), T1 (post-neoadjuvant), T2 (post surgery) and T3 (post-adjuvant therapy). Non-MPR, group of patients did not achieve pathologic response of MPR/pCR. (B) Mutational landscape of 12 samples at baseline (T0). (C) Comparison of positive rates from T0 to T3 between group of MPR/pCR and non-MPR. (D) Maximum variant allele frequency (mVAF) in group of non-MPR, MPR/pCR before and after neoadjuvant therapy and comparison of the change of mVAF. ctDNA, circulating tumor DNA; MPR, major pathological reactions; pCR, pathological reactions.

Fifteen patients with available plasma samples were enrolled for ctDNA analysis. In the 12 baseline samples (T0), ctDNA was detected in all patients (figure 6A). The positive rates decreased to 73.3%, 28.6% after neoadjuvant therapy and surgery and increased to 38.5% after adjuvant therapy. The mutational landscape at T0 revealed an average of three mutations per patient (figure 6B). *TP53* (75%), *CTNNB1* (33%), *TERT* (33%) were the top three altered genes.

When comparing patients with pathological response of MPR/pCR and non-MPR (non-MPR), we observed more mutations (counts of mutations in ctDNA at baseline) in MPR/pCR at baseline (6 mutations vs 2.5 mutations, p=0.025) (online supplemental figure S5A). After neoadjuvant therapy, a higher positive rate in group non-MPR (83.3% vs 33.3%, p=0.080) was revealed (figure 6C), and it re-increased to 50% after adjuvant therapy in non-MPR group (50% vs 0% in MPR/pCR, p=0.119).

Change of maximum variant allele frequency (mVAF) before and after neoadjuvant therapy was shown in figure 6D. Average mVAF decreased from 2.85% to 1.12% in patients with non-MPR (p=0.083) and decreased from 11.11% to 0.30% in patients with MPR/pCR (p=0.30).

### Association between ctDNA with R0 resection and RPS

Among patients with R0 resection and available ctDNA at T2 post surgery, 16.7% (2/12) was confirmed with ctDNA positive. This was significantly lower than that of patients with R1 resection (100%, p=0.016) (online supplemental figure S5B). This further confirmed minimal residue disease in patients who did not have radical resection.

We further explored the association between RFS and status of ctDNA. Patients with positive ctDNA after adjuvant therapy (T3) presented a trend of shorter RFS than those with negative ctDNA (mean Disease Free Survival(mDFS), 205.0 days vs not reached, p=0.170) (online supplemental figure S6–C). The association between change of ctDNA status and RFS during neoadjuvant, surgery and adjuvant therapy was analyzed, respectively (online supplemental figure S7A–C). The results showed that patients with favorable ctDNA change during adjuvant therapy (T2 positive/negative, T3 negative) could achieve superior RFS than those without ctDNA clearance (T2 positive/negative, T3 positive) (not reached vs 165.5 days, p<0.001).

**Figure 7:**
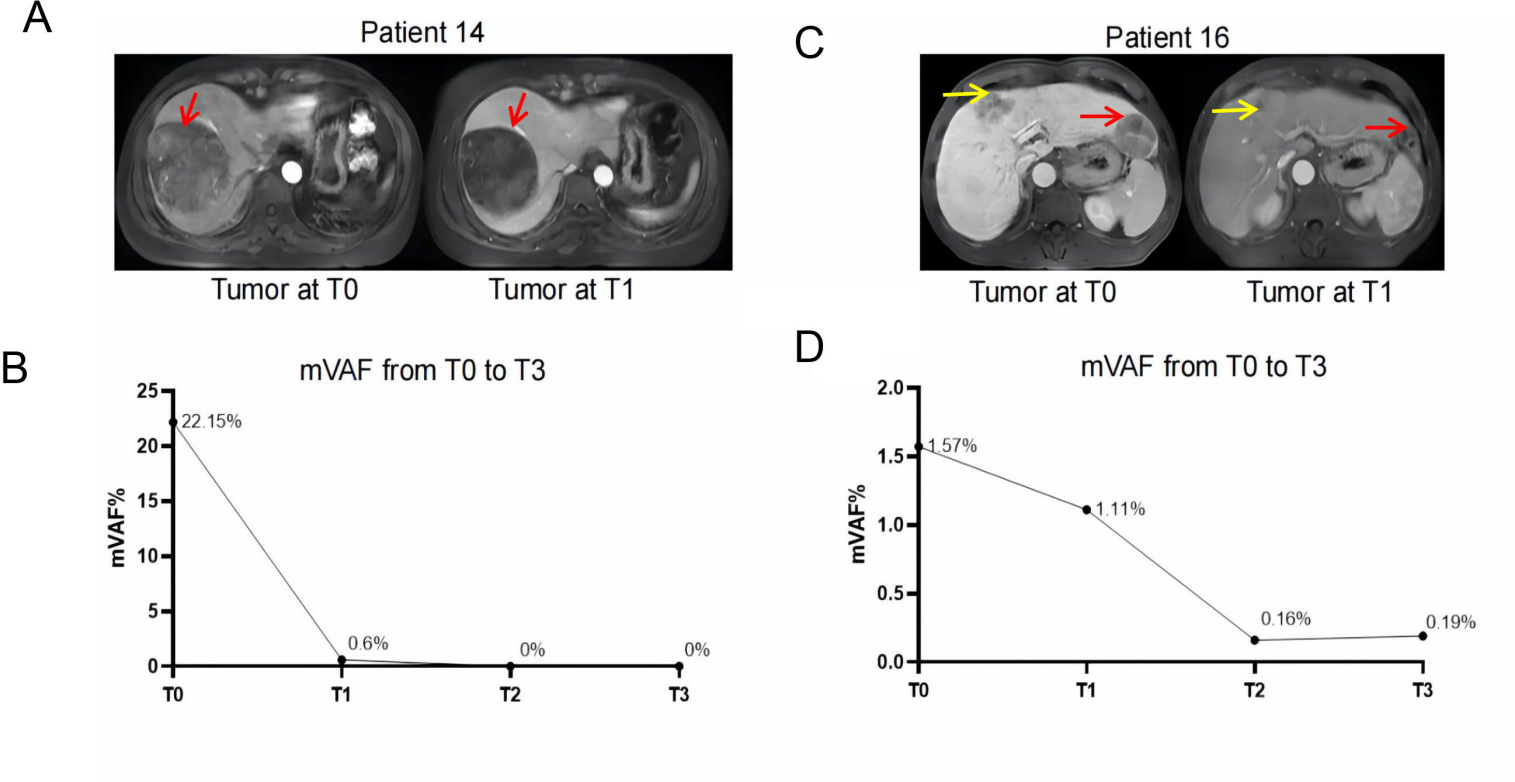
Change of tumor lesion and mVAF of ctDNA in Patient 14 (A–B) and 16 (C–D). Arrows represent lesions. mVAF, maximum variant allele frequency.

Change of ctDNA along with treatment in patient 14 and 16 was presented in figure 7. Patient 14 achieved MPR after neoadjuvant therapy and was relapse free after 1 year’s observation post surgery. Her mVAF had a 97.29%’s decrease at T1 and kept negative at T2 and T3 (figure 7A, B). As for Patient 16, his mVAF had a 29.30%’s decrease with a pathological response of SD (non-MPR) (figure 7C, D). After adjuvant therapy, his mVAF showed a slightly increase (0.16% to 0.19% from T2 to T3), relapse was confirmed at 190 days post surgery.

### The difference in proteomics between the responding and non-responding lesions

**Figure 8:**
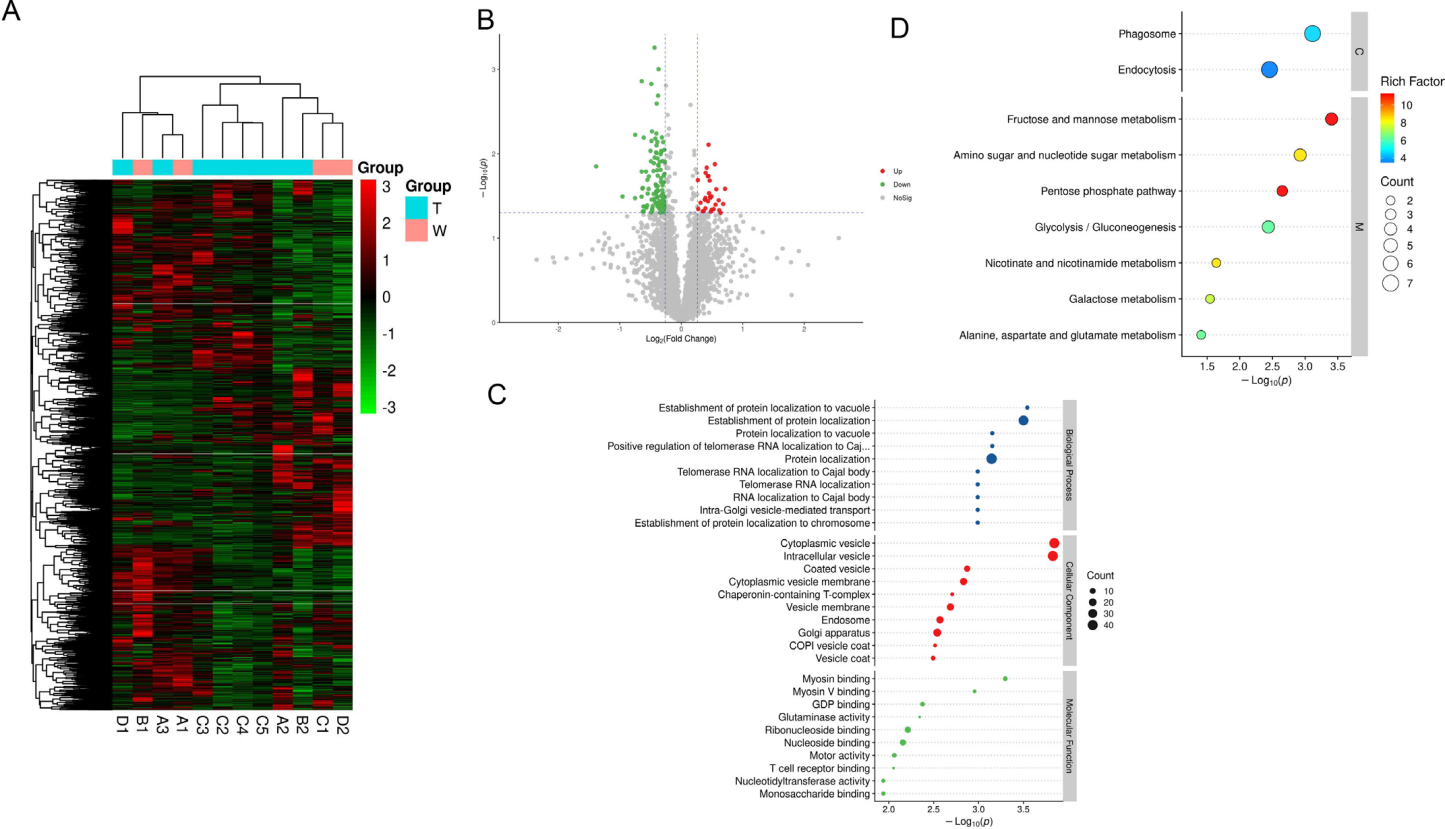
The difference in proteomics between the responding and non-responding tumors. (A–B) Heatmap (A) and volcano map (B) of different proteins between responding and non-responding lesions. T indicates non-responding lesions and W indicates responding lesions. (C) Gene ontology analysis results of different proteins between responding and non-responding lesions. (D) Kyoto Encyclopedia of Genes and Genomes pathway analysis of different proteins between responding and non-responding lesions.

Based on the above study results, the sensitivity of different lesions to neoadjuvant therapy was explored. Twelve neoadjuvant resection lesions (4 lesions were responding (R) with ≥50% tumor necrosis, 8 lesions were non-responding (NR) with <50% tumor necrosis) were collected and performed proteomics profiling. We were surprised to find that *PCBD1, TPI1, C1QA, FLAD1* and so on were significantly upregulated in the non-responding group compared with the responding group, while *FASN, TCP1, PKM* and so on were significantly upregulated in the responding group, which was shown by heatmap (figure 8A, online supplemental figure S8) and volcano map (figure 8B). GO analysis results showed that altered genes were significantly enriched in ‘protein location’, ‘Golgi apparatus’ and ‘T cell receptor binding’ and so on (figure 8C). KEGG pathway analysis showed that these altered genes were highly correlated with ‘glycolysis’ and ‘mannose metabolism’ and so on (figure 8D).

We also performed KEGG analysis on the proteins of the reactive lesions and non-reactive lesions of each patient, and the results of all four patients have been shown in online supplemental figure S9–D. Both the analysis of each patient and the common analysis of all samples suggested that abnormal glucose metabolism in patients with multifocal HCC might be related to different sensitivity of treatment in different lesions, but the underlying mechanisms needs to be verified by further studies.

## Discussion

This single-arm, open-label, phase II trial was designed to study the perioperative efficacy and safety of camrelizumab in combination with apatinib in patients with surgically resectable HCC. Our results showed that 16.7% patients with HCC reached ORR based on RECIST V.1.1 criteria, 33.3% patients with HCC achieved ORR based on mRECIST criteria, 17.6% patients with HCC achieved MPR, and 5.9% patients with HCC achieved pCR. Previous studies illustrated that the combination of immunotherapy with anti-angiogenesis also displayed encouraging efficacy with few safety issues in the treatment of patients with HCC in adjuvant setting. For instance, the combination therapy of avelumab and axitinib achieved 13.6% ORR in patients with advanced HCC according to RECIST 1.1% and 31.8% ORR according to mRECIST criteria.^43^Another open-label, phase II clinical trial exhibits that patients with advanced HCC reached the ORR of 34.3% and 23.8% after receiving camrelizumab plus apatinib as first-line therapy and second-line therapy based on RECIST V.1.1, respectively.^23^ However, our research is aimed at patients with resectable HCC, and the purpose of the research is completely different from the above two researches. So far, only Won Jin Ho’s study reported neoadjuvant cabozantinib and nivolumab could convert locally advanced HCC into resectable disease with enhanced antitumor immunity.^19^ Although their pathological response rate (42%, 5/12) was higher than our result (23.5%, 4/17), the types of patients with HCC we enrolled were different. In Won Jin Ho’s study, only 20% of patients with HBV-infected HCC were enrolled, while in our study, 83.3% of patients with HBV-infected HCC were enrolled, which is more suitable for the applicability of therapeutic effects for patients with HCC in Chinese population. In addition, our course of neoadjuvant therapy was significantly shorter than Won Jin Ho’s plan ^19^, because we pay more attention to the safety of preoperative neoadjuvant therapy, based on the fact that some scholars have reported severe cases of postoperative liver failure and death caused by preoperative immunotherapy plus targeted therapy.^44^ Therefore, shorter courses and smaller doses can ensure the safety of treatment. The advantage of preoperative immune neoadjuvant therapies is that it can induce a stronger immune response by taking advantage of the high preoperative tumor load. Based on this, we can also achieve a fairly satisfactory low recurrence rate by combining preoperative neoadjuvant therapy with postoperative adjuvant therapy.

A number of studies have reported a high postoperative recurrence rate of BCLC stage C patients, with a 1 year recurrence rate of more than 50%. Torzilli G^45^ and Yang T^46^ reported 1 year recurrence rates of 54% and 51.8%, respectively. In our research, the 1 year recurrence rate of single lesion patients with HCC in stage BCLC C treated by our regimen was only 22.2% (2/8), and that in patients with total HCC was 46.15%, lower than previous reports.^45 46^ Therefore, this protocol has achieved good results for patients with HCC especially for those with single foci. In Won Jin Ho’s study, among the seven patients who did not reach MPR/pCR,^19^ four patients recurred within 1 year, with a recurrence rate of 57%. In our study, among nine patients who did not reach MPR/pCR, five patients recurred within 1 year, with a recurrence rate of 55%. As for the recurrence time, the recurrence time of patients in Won Jin Ho’s study were 56–155 days, while ours was 127/148/205/293/326 days, indicating that the recurrence time was significantly prolonged. Based on the above information, we believe that a small dose of neoadjuvant therapy combined with postoperative assistance played a role in inhibiting recurrence and postoperative adjuvant therapy is very necessary in improving the low MPR/pCR rate brought by low-intensity neoadjuvant therapy. For multiple lesions, neoadjuvant regiment of camrelizumab with apatinib may not significantly reduce postoperative recurrence rates. In addition, we found that tumor diameter was significantly higher in the non-pCR group than in the pCR group, which might be explained by previous reports that small tumor foci has more immune cell infiltration and immune pathway upregulation compared with large tumor foci.^47^

Apart from the short-term efficacy, the safety of camrelizumab plus apatinib during perioperative period in patients with HCC is of great concern. In the present study, we observed that the proportion of grade 3 or 4 AEs in patients with surgical resectable HCC was 16.7% before surgery and 38.5% after surgery, no new AE was reported with all AEs being manageable and no death occurred during the perioperative period. These data reminds clinicians to closely monitor patients’ AE occurrence and reduce the dosage of camrelizumab plus apatinib or discontinue this therapy. In addition, we found that the mean value of duration of surgical resection was 248.0±77.3 min with a mean bleeding volume of 784.7±605.1 mL. The time for postoperative hospital was 13.2±4.2 days. The postoperative biliary leakage rate was 17.6%. These values were numerically higher than that in those patients with HCC who were not treated by the neoadjuvant regimen,^48^ while similar with that in those patients with HCC who received the neoadjuvant radiotherapy.^49^ This phenomenon might be explained as the surgical resection became more harder (manifesting as the increased bleeding volume and duration of surgical resection during the surgical resection) after the neoadjuvant therapy with immunotherapy plus anti-angiogenesis therapy, which might because of the administration of TKI. Furthermore, no death and no complications requiring reoperation occurred during the perioperative period. We discontinued the apatinib 3 weeks before the surgical resection. Unfortunately, even though we had fully considered the extension of the preoperative discontinuation of the TKI, the surgical resection was still hard and the postoperative biliary leakage rate was still high. These findings implied that a more sufficient preparation and a more dedicated manipulation during the perioperative period were needed to reduce the postoperative complications.

The biggest highlight of this study is that we found highly expressed DCs can predict good sensitivity to neoadjuvant therapy and the higher the DCs after neoadjuvant therapy, the less likely patients are to relapse based on TIME analysis. Previous studies had found that DCs may be involved in tumor immune checkpoint blockade (ICB) therapy. Enhanced DCs may increase responsiveness to ICB regimens through effector T cell activity regulation, epigenetic modulation and other factors.^50–52^ It is worth mentioning that our study is the first to report that the high expression of DC after neoadjuvant therapy predicts a better prognosis of patients, which is exactly in line with our treatment plan and explains why immunotherapy-based adjuvant therapy is still needed after surgical treatment. The high expression of DC after neoadjuvant therapy leading to more sensitive of postoperative adjuvant therapy, so patients are less likely to relapse. However, the sample size of our study is small, and a larger sample size is needed to conduct to support our point of view.

Our results of ctDNA revealed a higher positive rate (100%) among patients with stage IIb–IIIa disease with the high depth of sequencing. The top mutated genes of *TP53, CTNNB1* and *TERT* was in consistent with previous reports,^53 54^ while the frequencies might be affected by the limited sample size. By reviewing studies in HCC, we could also notice that, ctDNA has showed potential in prediction of treatment and prognostic outcome and disease monitoring.^55–57^ Currently, its value in predicting the efficacy of immunotherapy in HCC is gaining attention as well. In unresectable HCC, the mutations detected and variation trend of ctDNA were found associated with response and PFS after immunotherapy.^58 59^ As for perioperative treatment, ctDNA also showed potential of reflecting pathological reaction and recurrence post surgery in multiple cancers.^60–62^ In our exploratory analysis, patients with more decrease of mVAF and higher mutation burden tended to achieve favorable pathological response. ctDNA positive post perioperative treatment was more often seen in patients without MPR and showed potential of predicting earlier recurrence. These findings preliminary confirmed the in predicting value of ctDNA in recurrence. It also provided evidence of ctDNA as a potential compose biomarker to predict pathological response and relapse after perioperative immunotherapy in HCC. It could probably assist in tumor assessment before surgery and the following patients’ management, thus improving the clinical outcome. Meanwhile, the application of ctDNA on immunotherapy of HCC faces challenges including appropriate detection techniques for ctDNA, interpretation of ctDNA results, improvement of clinical outcome guided by ctDNA, which require further investigation.^63–65^

Based on the samples of patients enrolled in this study, we not only interpreted the sample differences before and after neoadjuvant therapy from the RNA (289 NanoString panel RNA sequencing) and DNA (ctDNA analysis) levels, but also explained the differential proteins of responding foci and non-responding foci from the protein level, which enriched our mechanism research level. We were pleasantly surprised to find that both the analysis of each patient and the common analysis of all samples suggested that abnormal glucose metabolism in patients with multifocal HCC may be related to different sensitivity of treatment in different lesions. Na Kwon Joong *et al* reported the ratio of GLUT3 and GLUT1, a surrogate of the reciprocal glucose metabolic activity between cancer and immune cells, predicts good immunotherapy response (anti-PD1 and anti-CTLA4).^66^ However, Renner Kathrin *et al* reported restricting glycolysis preserved T cell effector functions and augments checkpoint therapy.^67^ More mechanistic studies are expected to further elucidate the causes of drug resistance to anti-PD1 therapy. In response to these findings, we conclude that heterogeneity of the immune microenvironment of multifocal HCC should be considered when different or combined immunotherapy is performed for patients with multifocal HCC. The individualized immunotherapy strategy of single drug or combined targeted drug therapy with immune checkpoint inhibitors is an important step for patients with multifocal HCC to obtain better clinical efficacy.^47 68^

There were some limitations in the current study. First, the sample size of our study was relatively small. Second, this study was a single-arm phase II clinical trial, which lacked the control group. Third, this treatment option required adjustment and improvement based on clinical experience. Fourth, the follow-up period in the current study was relatively short (around 1 year). Last but not least, despite the reasons for drug sensitivity and resistance of neoadjuvant therapy have been evaluated from RNA, DNA and protein levels, our study still remained in the interpretation of phenomena, and further mechanism researches are needed. It is expected that the sequencing results of our study will lay the foundation for further research and open a bright light for further elucidation of the mechanism of camrelizumab plus apatinib therapy in HCC.

In conclusion, perioperative camrelizumab plus apatinib displays a promising efficacy and manageable toxicity in patients with resectable HCC. DCs infiltration might be a predictive marker of response to camrelizumab and apatinib as well as recurrence. ctDNA as a compose biomarker can predict pathological response and relapse. Abnormal glucose metabolism in patients with multifocal HCC may be related to different sensitivity of treatment in different lesions.

## Data Availability

Data sharing not applicable as no datasets generated and/or analysed for this study.
