# Supplementary material for: Efficacy and safety of camrelizumab plus apatinib during the perioperative period in resectable hepatocellular carcinoma: a single-arm, open label, phase II clinical trial
Source: J Immunother Cancer. 2022 Apr 1;10(4):e004656. doi: 10.1136/jitc-2022-004656 (PMC8981365; doi:10.1136/jitc-2022-004656)
Supplement: Supplementary data [file jitc-2022-004656supp001.pdf]

Table S1 Baseline patient demographics and disease characteristics

**Characteristic**	N=18
Median age, years (range)	54.7(34,76)
Sex (male/female), *n*	17/1
ECOG performance status (0/1), *n*	18/0
Etiology of HCC (HBV/HCV/non-viral), *n*	15/1/2
BCLC stage (B/C), *n*	5/13
China liver cancer stage (IIb/IIIa), *n*	5/13
Child-Pugh class (A5/A6), *n*	12/6
Baseline AFP ≥400 ng/mL, *n* (%)	8(44.4%)

ECOG, Eastern Cooperative Oncology Group; HCC, hepatocellular carcinoma; HBV, Hepatitis B virus; HCV, Hepatitis C virus; BCLC, Barcelona Clinic Liver Cancer; AFP, alpha-fetoprotein.

Table S2 Treatment response of HCC patients after neoadjuvant therapy

**Variable**	**RECIST 1.1**	**mRECIST**
Best overall response, n (%)		
CR	0	0
PR	3	6
SD	14	11
PD	1	1
Unidentified	0	0
ORR (CR+PR),% (n/N)	16.7	33.3
95% CI	4.4-42.3	14.4-58.8
DCR, % (n/N)	94.4	94.4
95% CI	70.6-99.7	70.6-99.7

CR, complete response; PR, partial response; SD, stable disease; PD, progressive disease; ORR, objective response rate; DCR, disease control rate; CI, confidence interval.

Table S3 Characteristics of surgical and postoperative features

Patient. No	Intrahepatictumor size, mm	Number of intrahepatic tumors	BCLC stage	CNLC stage	Tumor response, by RECIST v1.1	Tumor response, by mRECIST	PHLF*	Postoperative complication	Postoperative hospital stay, days	MPR/pCR	RFS, days
1	87.4	1	C	IIIa	SD	PR	N	biliary leakage	17	MPR	555
2	43.5	>3	B	IIb	SD	PR	N	N	9	N	64
3	130.7	1	C	IIIa	SD	SD	Y	N	11	N	449
4	133.8	1	C	IIIa	SD	SD	Y	N	15	MPR	275
5	32.6	>3	B	IIb	PR	PR	N	N	7	pCR	421
6	81.2	>3	B	IIb	PR	PR	Y	anemia	19	N	326
7	30.5	1	C	IIIa	SD	SD	N	anemia	15	N	430
8	126.5	1	C	IIIa	SD	SD	N	N	9	N	411
9	82.5	2	C	IIIa	SD	SD	N	N	10	N	-
10	87.2	>3	C	IIIa	SD	SD	N	N	7	N	418
11	104.5	1	C	IIIa	SD	SD	Y	biliary leakage	16	N	127

12	80.0	>3	B	IIb	SD	SD	N	N	9	N	293
13	135.5	2	C	IIIa	SD	SD	Y	N	15	N	148
14	108.1	1	C	IIIa	SD	PR	N	N	14	MPR	402
15	78.1	1	C	IIIa	SD	SD	N	biliary leakag e	17	N	380
16	43.4	>3	B	IIb	PR	PR	Y	ACS	21	N	205
17	101.6	1	C	IIIa	PD	PD	-	-	-	-	-
18	40.6	2	C	IIIa	SD	SD	Y	N	14	N	-

PHLF, post-hepatectomy liver failure; pCR, pathological complete response; *Classified according to the International Study Group of Liver Surgery; ACS, acute coronary syndrome.

Table S4 Number of individuals with treatment related AEs during the neoadjuvant treatment period

**Toxicity (n=18) (n, %)**	**All grades**	**1/2 grade**	**3/4 grade**
All	16 (88.9%)	14 (77.8%)	3 (16.7%)
**Systemic disease**			
Fever	7 (38.9%)	7 (38.9%)	0
Fatigue	2 (11.1%)	2 (11.1%)	0
**Cardio-renal**			
Hypertension	6 (33.3%)	5 (27.8%)	1 (5.6%)
**Liver Disease**			
LDH increased	6 (33.3%)	6 (33.3%)	0
Blood bilirubin increased	5 (27.8%)	5 (27.8%)	
AST increased	4 (22.2%)	4 (22.2%)	0
ALP increased	4 (22.2%)	4 (22.2%)	0
GGT increased	2 (11.1%)	2 (11.1%)	0
ALT increased	1 (5.6%)	1 (5.6%)	0
Drug-induced liver damage	1 (5.6%)	0 (0%)	1 (5.6%)
**Coagulation**			
APTT prolonged	6 (33.3%)	6 (33.3%)	0
Gastrointestinal			
Abdominal pain	6 (33.3%)	6 (33.3%)	0
Periodontal disease	1 (5.6%)	1 (5.6%)	0
**Dermatologic**			
Rash	3 (16.7%)	1 (5.6%)	2 (11.1%)
Palmar-plantar erythrodysesthesia syndrome	1 (5.6%)	1 (5.6%)	0
**Kidney and urinary system**			
Proteinuria	3 (16.7%)	3 (16.7%)	0
**Respiratory system**			
Cough	1 (5.6%)	1 (5.6%)	0
**Blood system**			
Neutropenia	3 (16.7%)	2 (11.1%)	1 (5.6%)
Thrombocytopenia	3 (16.7%)	3 (16.7%)	0
Anemia	2 (11.1%)	2 (11.1%)	0
Leukopenia	2 (11.1%)	2 (11.1%)	0
Hypoalbuminemia	3 (16.7%)	3 (16.7%)	0

AE, adverse event; LDH, lactate dehydrogenase; AST, aspartate aminotransferase; ALP, alkaline phosphatase; GGT, gamma-glutamyl transferase; ALT,

alanine aminotransferase; APTT, activated partial thromboplastin time.

Table S5 Characteristics of surgical and postoperative features

Patient. No	Intrahepatictumor size, mm	Number of intrahepatic tumors	BCLC stage	CNLC stage	Tumor response, by RECIST v1.1	Tumor response, by mRECIST	PHLF *	Postoperative complication	Postoperative hospital stay, days	MPR /pCR	PFS, days
001	87.4	1	C	IIIa	SD	PR	N	biliary leakage	17	MPR	555
002	43.5	>3	B	IIb	SD	PR	N	N	9	N	64
003	130.7	1	C	IIIa	SD	SD	Y	N	11	N	449
004	133.8	1	C	IIIa	SD	SD	Y	N	15	MPR	275
005	32.6	>3	B	IIb	PR	PR	N	N	7	pCR	421
006	81.2	>3	B	IIb	PR	PR	Y	anemia	19	N	326
007	30.5	1	C	IIIa	SD	SD	N	anemia	15	N	430
008	126.5	1	C	IIIa	SD	SD	N	N	9	N	411
009	82.5	2	C	IIIa	SD	SD	N	N	10	N	-
010	87.2	>3	C	IIIa	SD	SD	N	N	7	N	418
011	104.5	1	C	IIIa	SD	SD	Y	biliary leakage	16	N	127
012	80.0	>3	B	IIb	SD	SD	N	N	9	N	293
013	135.5	2	C	IIIa	SD	SD	Y	N	15	N	148
014	108.1	1	C	IIIa	SD	PR	N	N	14	MPR	402
015	78.1	1	C	IIIa	SD	SD	N	biliary leakage	17	N	380
016	43.4	>3	B	IIb	PR	PR	Y	ACS	21	N	205
017	101.6	1	C	IIIa	PD	PD	-	-	-	-	-
018	40.6	2	C	IIIa	SD	SD	Y	N	14	N	-

PHLF, post-hepatectomy liver failure; pCR, pathological complete response; MPR, major pathological reaction; PFS, progression-free survival; ACS, acute coronary syndrome. *Classified according to the International Study Group of Liver Surgery.

Table S6 Number of individuals with treatment related AEs during the adjuvant treatment period

Toxicity (n=13) (n, %)	All grades	1/2 grade	3/4 grade
All	13 (100 %)	12 (92.3%)	5 (38.5 %)
**Cardio-renal**			
Hypertension	9 (69.2%)	6 (46.2%)	3 (23.1%)
**Liver Disease**			
ALP increased	2 (15.4%)	1 (7.7%)	1 (7.7%)
GGT increased	3 (23.1%)	2 (15.4%)	1 (7.7%)
Blood bilirubin increased	2 (15.4%)	2 (15.4%)	0
ALT increased	2 (15.4%)	2 (15.4%)	0
AST increased	1 (7.7%)	1 (7.7%)	0
**Kidney and urinary system**			
Proteinuria	6 (46.2%)	6 (46.2%)	0
**Endocrine System**			
TSH increased	6 (46.2%)	6 (46.2%)	0

Hypothyroidism	2 (15.4%)	2 (15.4%)	0
**Gastrointestinal**			
Diarrhea	1 (7.7%)	0	1 (7.7%)
Periodontal disease	2 (15.4%)	2 (15.4%)	0
Abdominal pain	1 (7.7%)	1 (7.7%)	0
Oral mucositis	1 (7.7%)	1 (7.7%)	0
Anorexia	1 (7.7%)	1 (7.7%)	0
**Systemic disease**			
Fatigue	2 (15.4%)	2 (15.4%)	0
**Dermatologic**			
RCCEP	3 (23.1%)	3 (23.1%)	0
Palmar-plantar erythrodysesthesia syndrome	1 (7.7%)	1 (7.7%)	0
**Others**			
Hyperuricemia	1 (7.7%)	1 (7.7%)	0
**Blood system**			
Leukopenia	7 (53.8%)	6 (46.2%)	1 (7.7%)
Neutropenia	7 (53.8%)	5 (33.3%)	2（15.4%)
Thrombocytopenia	5 (38.5%)	4 (30.8%)	1 (7.7%)
Anemia	2 (15.4%)	2 (15.4%)	0
Hypoalbuminemia	1 (7.7%)	1 (7.7%)	0

ALP, alkaline phosphatase; GGT, gamma-glutamyl transferase; ALT, alanine aminotransferase; AST, aspartate aminotransferase; TSH, thyroid-stimulating hormone; RCCEP, reactive cutaneous capillary endothelial proliferation.

Supplementary Figures

Figure S1. CT scan changes of patients from pre-treatment to before surgery.

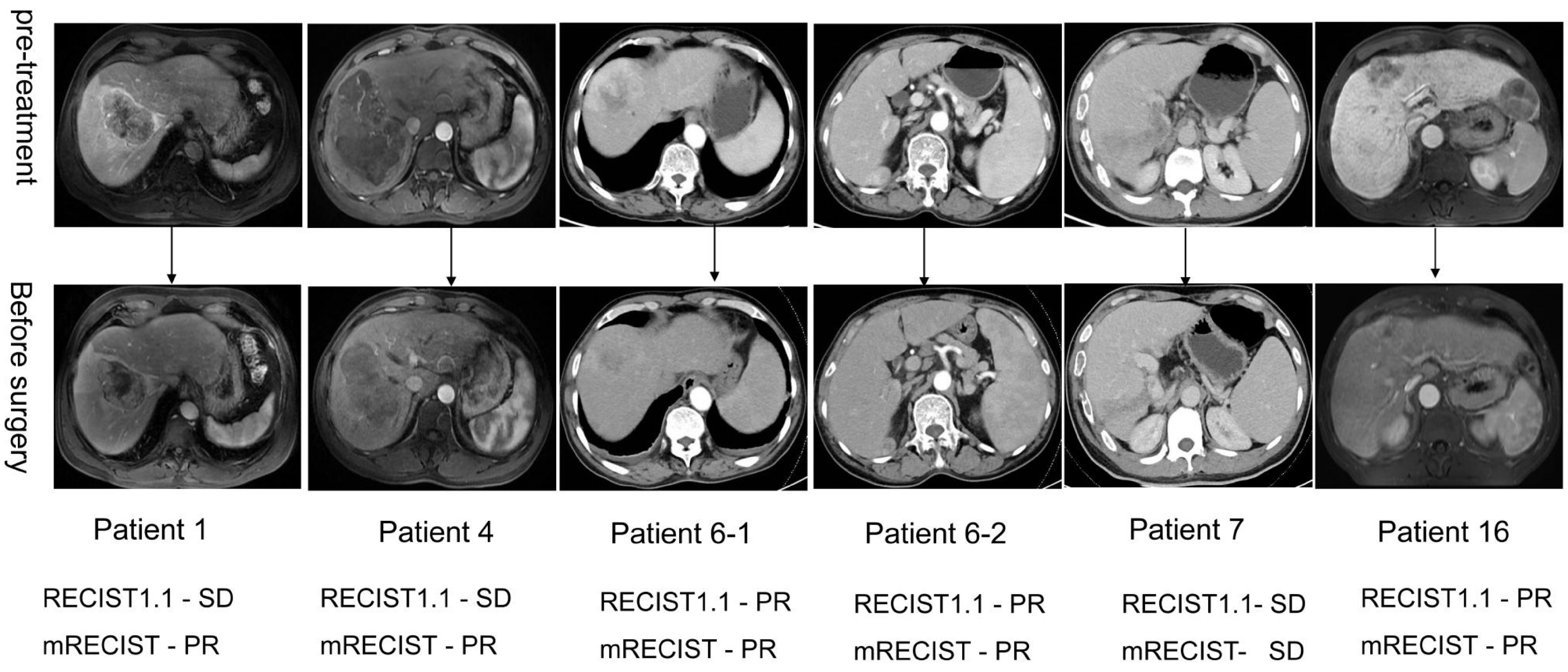

Figure S2. Patient 17; male; 45 years old; IIIa; diameter 101.6mm; PD was evaluated after neoadjuvant, tumor thrombi was formed in vena cava; surgery was not performed, radiotherapy was added on the basis of camrelizumab+apatinib, tumor thrombi disappeared, TACE was further added, tumor also decreased, PR was evaluated, surgical treatment could be performed. But the patient refused surgery and continued systematic treatment. Arrows represent tumor thrombus.

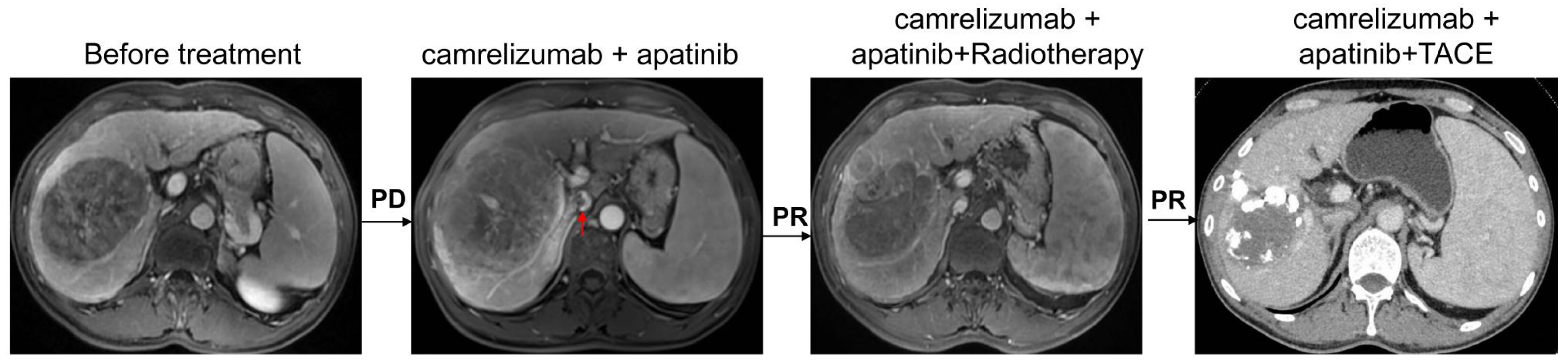

Patient 17

Figure S3. Patient follow-up. (**A**) The 1-year RFS rate was 53.85% (95% CI: 24.77%-75.99%). (**B**) The RFS of patients with or without MPR/pCR. (**C**) The RFS of patients with multiple foci and single foci .

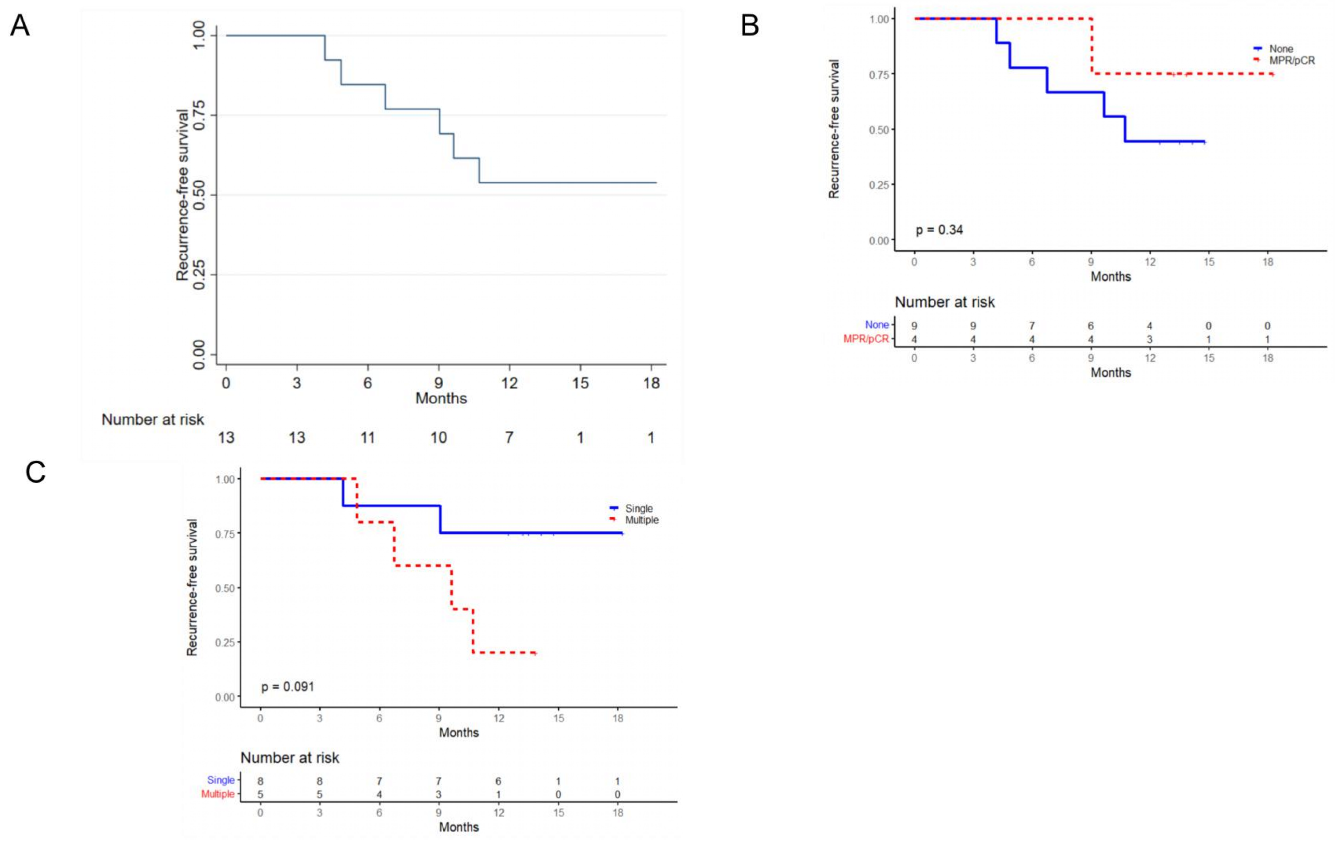

Figure S4. (**A-B**) The relationship between the DC (**A**) and IFNγ (**B**) of the post-treatment samples and the recurrence risk in single lesion patients. (**C-D**) The relationship between the DC (**C**) and IFNγ (**D**) of the post-treatment samples and the recurrence risk in multi and single lesion patients.

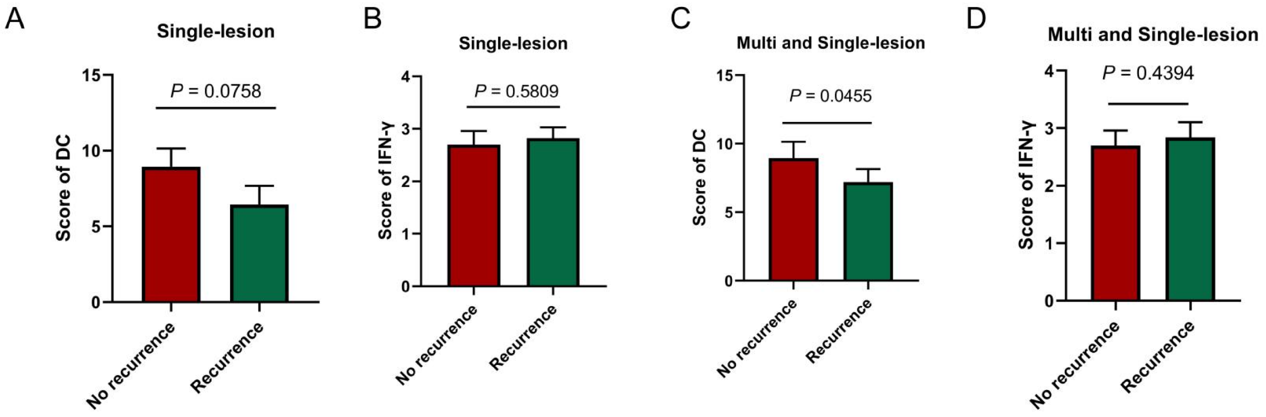

Figure S5. (**A**) Comparison of mutations detected in ctDNA at baseline (T0) between group of non-MPR (n=10) and MPR (n=2). (**B**) Comparison of positive rates of ctDNA post-surgery (T2) between patients with R1 (n=2) and R0 (n=12) resection.

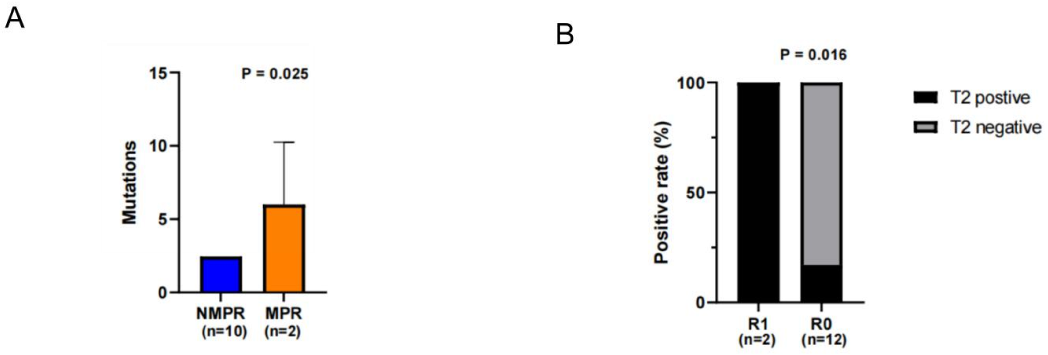

Figure S6. Kaplan-Meier curve of relapse free survival (RFS) with positive and negative status of ctDNA at T1 (**A**), T2 (**B**), and T3 (**C**) respectively.

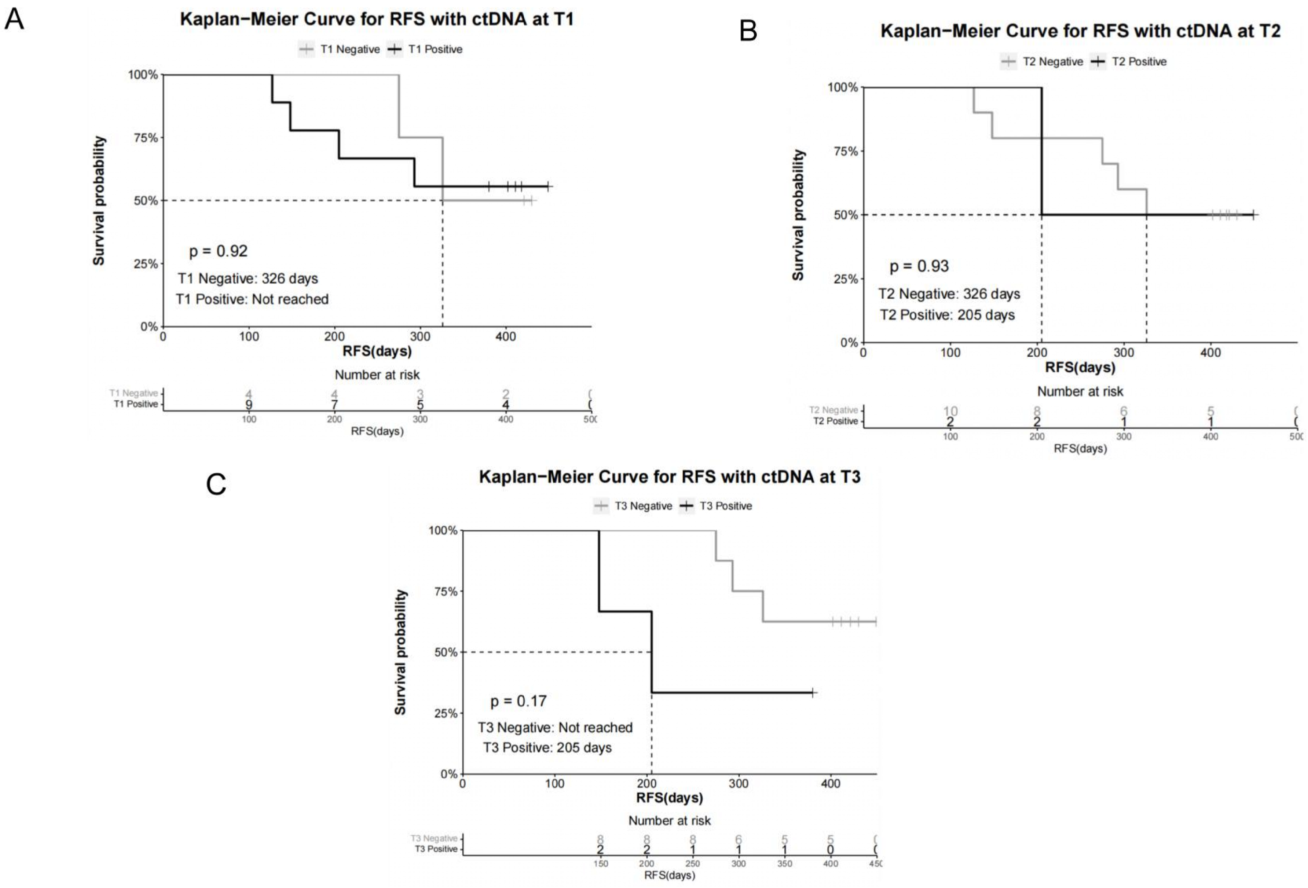

Figure S7. Kaplan-Meier curve of relapse free survival (RFS) with change of ctDNA status before and after neoadjuvant (T0 to T1) (**A**), surgery (T1 to T2) (**B**), and adjuvant therapy (T2 to T3) (**C**) respectively. Favorable ctDNA change was defined as positive to negative or remaining negative. Unfavorable ctDNA change was accordingly defined as positive/negative to positive.

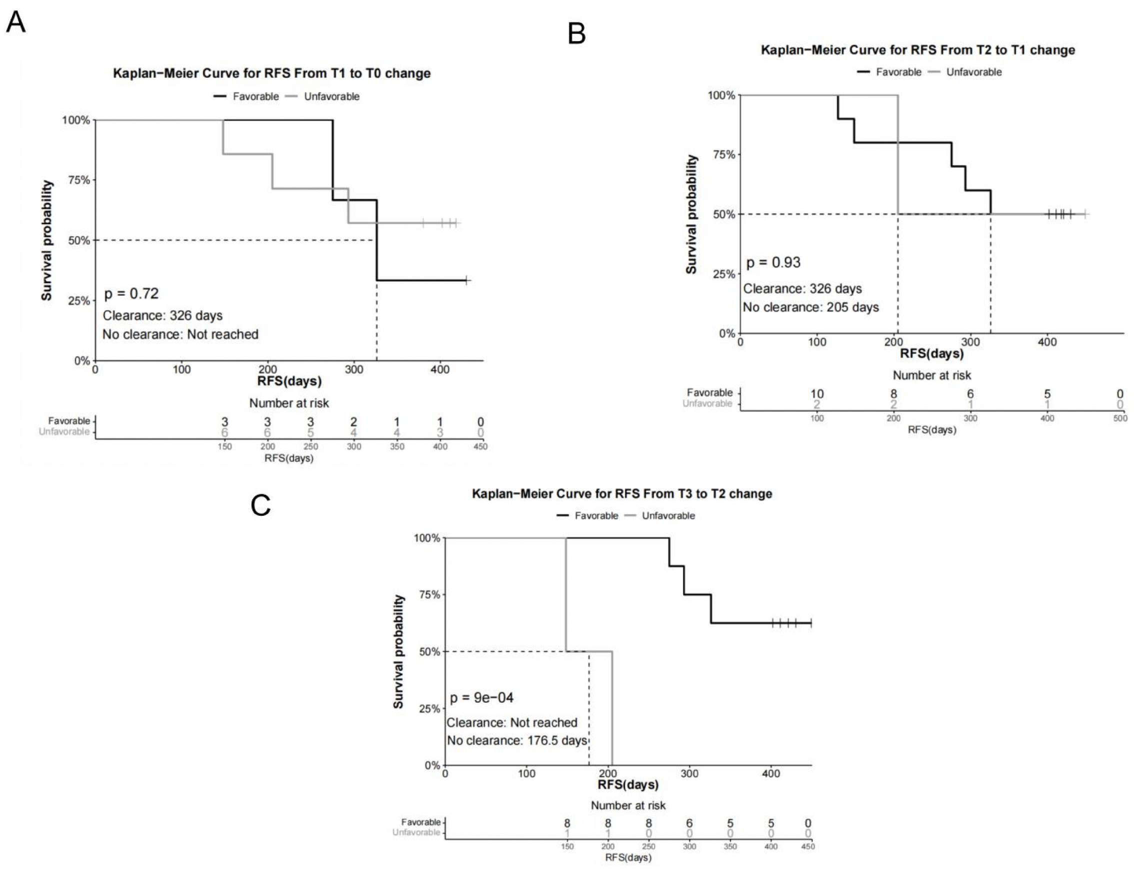

Figure S8. Heatmap listing specific differential genes.

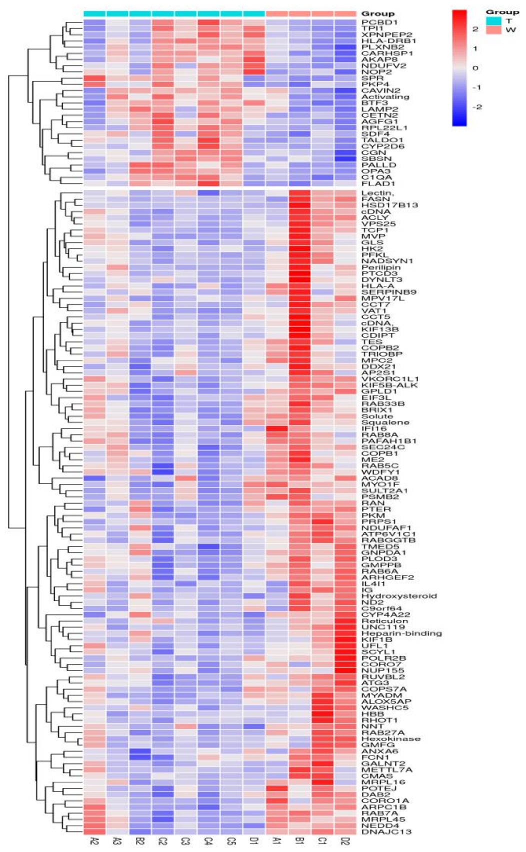

Figure S9. KEGG analysis on the proteins of the reactive lesions and non-reactive lesions of each patient. **A-D** represents four patients.

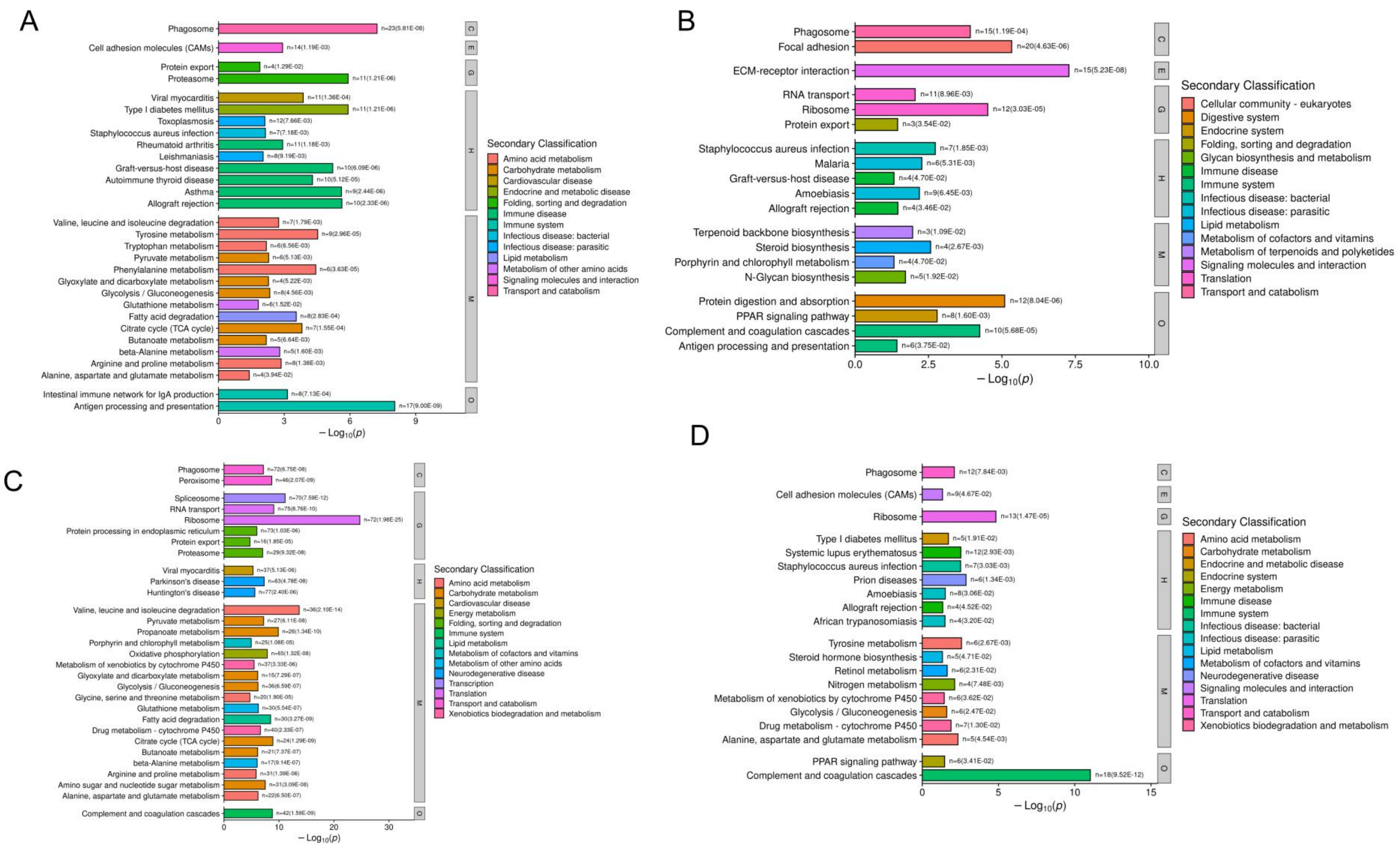
A
Phagosome n=23(5.81E-08)
Cell adhesion molecules (CAMs) n=14(1.19E-03)
Protein export n=4(1.29E-02)
Proteasome n=11(1.21E-06)
Viral myocarditis n=11(1.36E-04)
Type I diabetes mellitus n=11(1.21E-06)
Toxoplasmosis n=12(7.66E-03)
Staphylococcus aureus infection n=7(7.18E-03)
Rheumatoid arthritis n=11(1.18E-03)
Leishmaniasis n=8(9.19E-03)
Graft-versus-host disease n=10(6.09E-06)
Autoimmune thyroid disease n=10(5.12E-05)
Asthma n=9(2.44E-06)
Allograft rejection n=10(2.33E-06)
Valine, leucine and isoleucine degradation n=7(1.79E-03)
Tyrosine metabolism n=9(2.96E-05)
Tryptophan metabolism n=6(6.56E-03)
Pyruvate metabolism n=6(5.13E-03)
Phenylalanine metabolism n=6(3.63E-05)
Glyoxylate and dicarboxylate metabolism n=4(5.22E-03)
Glycolysis / Gluconeogenesis n=8(4.56E-03)
Glutathione metabolism n=6(1.52E-02)
Fatty acid degradation n=8(2.83E-04)
Citrate cycle (TCA cycle) n=7(1.55E-04)
Butanoate metabolism n=5(6.64E-03)
beta-Alanine metabolism n=5(1.60E-03)
Arginine and proline metabolism n=8(1.38E-03)
Alanine, aspartate and glutamate metabolism n=4(3.94E-02)
Intestinal immune network for IgA production n=8(7.13E-04)
Antigen processing and presentation n=17(9.00E-09)
C
E
G
H
M
O
0
3
6
9
− Log10(p)
Secondary Classification
Amino acid metabolism
Carbohydrate metabolism
Cardiovascular disease
Endocrine and metabolic disease
Folding, sorting and degradation
Immune disease
Immune system
Infectious disease: bacterial
Infectious disease: parasitic
Lipid metabolism
Metabolism of other amino acids
Signaling molecules and interaction
Transport and catabolism
B
Phagosome n=15(1.19E-04)
Focal adhesion n=20(4.63E-06)
ECM-receptor interaction n=15(5.23E-08)
RNA transport n=11(8.96E-03)
Ribosome n=12(3.03E-05)
Protein export n=3(3.54E-02)
Staphylococcus aureus infection n=7(1.85E-03)
Malaria n=6(5.31E-03)
Graft-versus-host disease n=4(4.70E-02)
Amoebiasis n=9(6.45E-03)
Allograft rejection n=4(3.46E-02)
Terpenoid backbone biosynthesis n=3(1.09E-02)
Steroid biosynthesis n=4(2.67E-03)
Porphyrin and chlorophyll metabolism n=4(4.70E-02)
N-Glycan biosynthesis n=5(1.92E-02)
Protein digestion and absorption n=12(8.04E-06)
PPAR signaling pathway n=8(1.60E-03)
Complement and coagulation cascades n=10(5.68E-05)
Antigen processing and presentation n=6(3.75E-02)
C
E
G
H
M
O
0.0
2.5
5.0
7.5
10.0
− Log10(p)
Secondary Classification
Cellular community - eukaryotes
Digestive system
Endocrine system
Folding, sorting and degradation
Glycan biosynthesis and metabolism
Immune disease
Immune system
Infectious disease: bacterial
Infectious disease: parasitic
Lipid metabolism
Metabolism of cofactors and vitamins
Metabolism of terpenoids and polyketides
Signaling molecules and interaction
Translation
Transport and catabolism
C
Phagosome n=72(6.75E-08)
Peroxisome n=46(2.07E-09)
Spliceosome n=70(7.59E-12)
RNA transport n=75(8.76E-10)
Ribosome n=72(1.98E-25)
Protein processing in endoplasmic reticulum n=73(1.03E-06)
Protein export n=16(1.85E-05)
Proteasome n=29(9.32E-08)
Viral myocarditis n=37(5.13E-06)
Parkinson's disease n=63(4.78E-08)
Huntington's disease n=77(2.40E-06)
Valine, leucine and isoleucine degradation n=36(2.10E-14)
Pyruvate metabolism n=27(6.11E-08)
Propanoate metabolism n=26(1.34E-10)
Porphyrin and chlorophyll metabolism n=25(1.08E-05)
Oxidative phosphorylation n=65(1.32E-08)
Metabolism of xenobiotics by cytochrome P450 n=37(3.33E-06)
Glyoxylate and dicarboxylate metabolism n=15(7.29E-07)
Glycolysis / Gluconeogenesis n=36(6.59E-07)
Glycine, serine and threonine metabolism n=20(1.90E-05)
Glutathione metabolism n=30(5.54E-07)
Fatty acid degradation n=30(3.27E-09)
Drug metabolism - cytochrome P450 n=40(2.33E-07)
Citrate cycle (TCA cycle) n=24(1.29E-09)
Butanoate metabolism n=21(7.37E-07)
beta-Alanine metabolism n=17(9.14E-07)
Arginine and proline metabolism n=31(1.39E-06)
Amino sugar and nucleotide sugar metabolism n=31(3.09E-08)
Alanine, aspartate and glutamate metabolism n=22(6.50E-07)
Complement and coagulation cascades n=42(1.59E-09)
C
G
H
M
O
0
10
20
30
− Log10(p)
Secondary Classification
Amino acid metabolism
Carbohydrate metabolism
Cardiovascular disease
Energy metabolism
Folding, sorting and degradation
Immune system
Lipid metabolism
Metabolism of cofactors and vitamins
Metabolism of other amino acids
Neurodegenerative disease
Transcription
Translation
Transport and catabolism
Xenobiotics biodegradation and metabolism
D
Phagosome n=12(7.84E-03)
Cell adhesion molecules (CAMs) n=9(4.67E-02)
Ribosome n=13(1.47E-05)
Type I diabetes mellitus n=5(1.91E-02)
Systemic lupus erythematosus n=12(2.93E-03)
Staphylococcus aureus infection n=7(3.03E-03)
Prion diseases n=6(1.34E-03)
Amoebiasis n=8(3.06E-02)
Allograft rejection n=4(4.52E-02)
African trypanosomiasis n=4(3.20E-02)
Tyrosine metabolism n=6(2.67E-03)
Steroid hormone biosynthesis n=5(4.71E-02)
Retinol metabolism n=6(2.31E-02)
Nitrogen metabolism n=4(7.48E-03)
Metabolism of xenobiotics by cytochrome P450 n=6(3.62E-02)
Glycolysis / Gluconeogenesis n=6(2.47E-02)
Drug metabolism - cytochrome P450 n=7(1.30E-02)
Alanine, aspartate and glutamate metabolism n=5(4.54E-03)
PPAR signaling pathway n=6(3.41E-02)
Complement and coagulation cascades n=18(9.52E-12)
C
E
G
H
M
O
0
5
10
15
− Log10(p)
Secondary Classification
Amino acid metabolism
Carbohydrate metabolism
Endocrine and metabolic disease
Endocrine system
Energy metabolism
Immune disease
Immune system
Infectious disease: bacterial
Infectious disease: parasitic
Lipid metabolism
Metabolism of cofactors and vitamins
Neurodegenerative disease
Signaling molecules and interaction
Translation
Transport and catabolism
Xenobiotics biodegradation and metabolism
